# Plant-Derived Exosomes as Novel Nanotherapeutics Contrive Glycolysis Reprogramming-Mediated Angiogenesis for Diabetic Ulcer Healing

**DOI:** 10.34133/bmr.0035

**Published:** 2024-06-05

**Authors:** Minhong Tan, Yuda Liu, Yang Xu, Ge Yan, Nan Zhou, Haoran Chen, Zhihong Jiang, Lihua Peng

**Affiliations:** ^1^ College of Pharmaceutical Sciences, Zhejiang University, Hangzhou 310058, PR China.; ^2^ School of Materials Science and Engineering, Zhejiang University, Hangzhou 310058, PR China.; ^3^ State Key Laboratory of Quality Research in Chinese Medicine, Macau University of Science and Technology, Macau, PR China.

## Abstract

Reversal of endothelial cell (EC) dysfunction under high-glucose (HG) conditions to achieve angiogenesis has remained a big challenge in diabetic ulcers. Herein, exosomes derived from medicinal plant ginseng (GExos) were shown as excellent nanotherapeutics with biomimetic cell membrane-like structures to be able to efficiently transfer the encapsulated active substances to ECs, resulting in a marked reprogramming of glycolysis by up-regulating anaerobic glycolysis and down-regulating oxidative stress, which further restore the proliferation, migration, and tubule formation abilities of ECs under HG conditions. In vivo, GExos enhance the angiogenesis and nascent vessel network reconstruction in full-thickness diabetic complicated skin ulcer wounds in mice with high biosafety. GExos were shown as promising nanotherapeutics in stimulating glycolysis reprogramming-mediated angiogenesis in diabetic ulcers, possessing wide application potential for reversing hyperglycemic dysangiogenesis and stimulating vascular regeneration.

## Introduction

The impaired angiogenesis induced by endothelial dysfunction typically promotes the non-healing characteristics in diabetic chronic ulcers. [1,2], where the disordered glucose metabolism and the resulting oxidative stress injury in hyperglycemia are key inducers [3,4]. Synthesized nanomaterials have been widely investigated as nanodrugs or delivery vehicles to enhance angiogenesis [5,6]. These approaches leverage the properties of nanomaterials to release angiogenic metal ions or utilize themselves as vehicles to deliver the incorporated active molecules [7–10]. However, most of these reported nanoplatforms face persistent challenges in slow or non-degradation-induced compromised biocompatibility or long-term biorisk concerns. The limited efficacy in loading and controlling external molecules release remains an obstacle to overcome [11,12]. Furthermore, up to now, few studies have reported the potential of nanoplatforms in pro-angiogenesis under high-glucose (HG) conditions, of which the reversal of endothelial cells’ (ECs’) functions is one of the most important preconditions for angiogenesis to treat hyperglycemic complications, such as diabetic ulcers.

Nowadays, exosomes derived from medicinal plants that naturally secrete bioactive nanotherapeutics with cell membrane-like structures and stable nanoscale size have been demonstrated for their exceptional biocompatibility, biodegradability, and capability to bypass biological barriers, possessing great potential as therapeutic nanoplatforms for various disease therapies [13–15]. For instance, numerous studies have illustrated that plant-derived exosomes demonstrate remarkable therapeutic effects, including restoring redox balance [16], anti-leukemic [17], anti-tumor [18] properties, and promoting tissue regeneration [19]. These effects may be attributed to the ability of exosomes to effectively deliver diverse therapeutic payloads, especially small RNAs, to a desired target and influence the diseased cells or tissues [20,21]. Moreover, the lipid and protein in the exosome’s membrane facilitate their cellular uptake and transport capacity, and the complete degradation feature minimizes the side effects and toxicity [22].

**Fig. 1.**
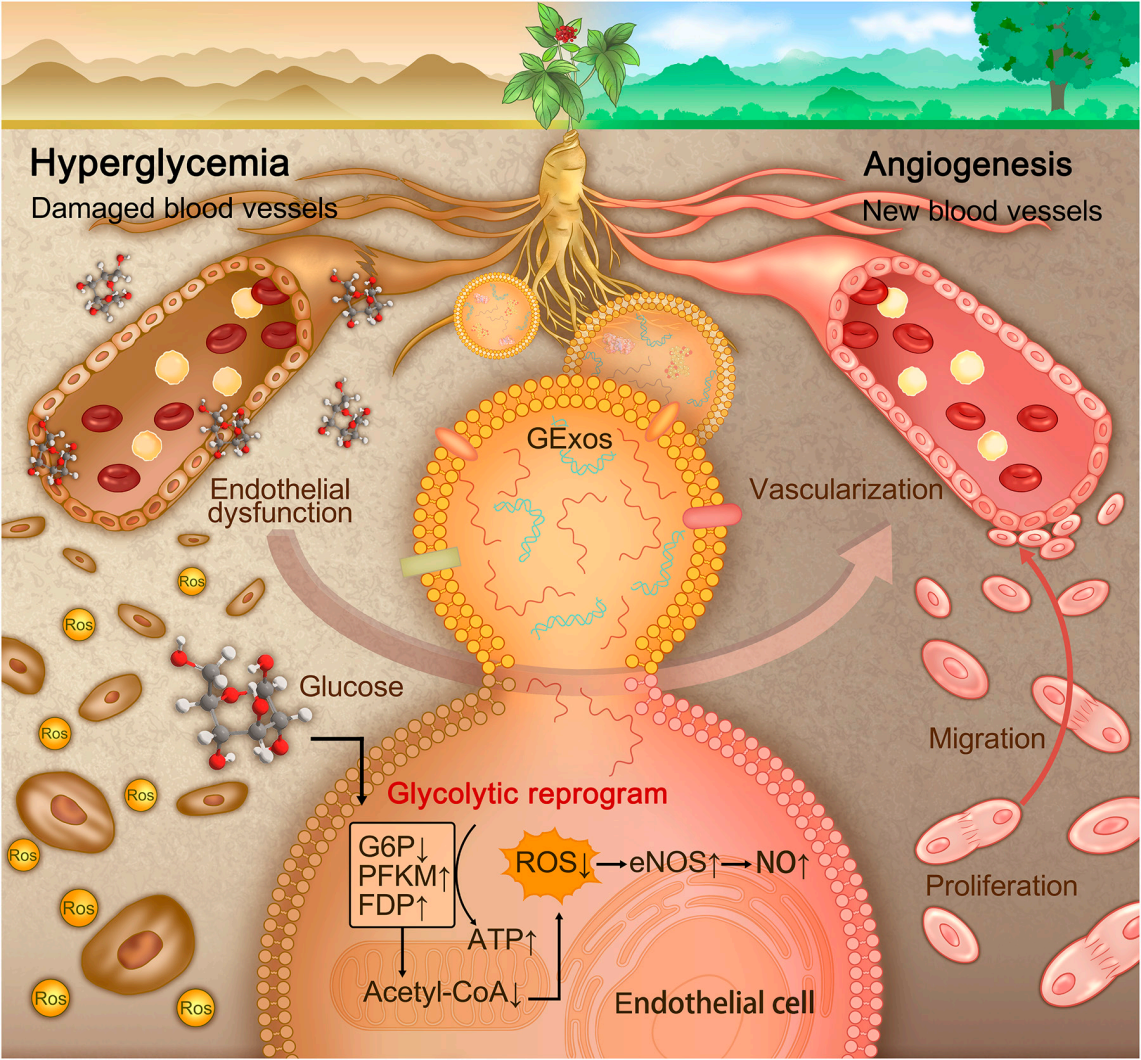
Scheme of the GExos stimulation in angiogenic activities of ECs under HG conditions by glycolytic reprogram mechanisms.

Ginseng is a medicinal plant widely used as a therapeutic drug, functional food, and nutrient agent for vascular diseases. Extensive studies and clinical usage have highlighted the stimulation of ginseng and its extracts in angiogenesis for various diseases including diabetic skin ulcers, of which non-angiogenesis is the biggest challenge for healing [23–27]. It is therefore hypothesized that ginseng-derived exosomes (GExos) may possess angiogenic effects for treating diabetic skin ulcers. Accordingly, herein, for the first time, the efficacy and potential mechanism of GExos as nanotherapeutics to transfer the incorporated substances into ECs to stimulate angiogenesis under hyperglycemic conditions for diabetic ulcer healing were investigated. Figure 1 illustrates the hypothesized potential of GExos in reversing the ECs’ functions in an HG environment for angiogenesis. Besides, the first evidence achieved in this study demonstrating the angiogenic mechanisms of GExos under hyperglycemic conditions through glycolysis reprogramming is also indicated.

## Materials and Methods

### Exosome isolation, characterization, and quantification

GExos were isolated from Ginseng root (provided by the Ginseng Base of Changbaishan Mountain, Jilin Province, China). Before the extraction of GExos, each batch of ginseng underwent pesticide content testing following the procedures outlined in the Chinese Pharmacopoeia. As Table S1 shows, either no pesticide residues or only trace amounts were detected in plant ginseng, meeting the limit standards of the Chinese Pharmacopoeia. Briefly, ginseng was washed and ground in a blender to obtain juice by differential centrifugation at 3,000 *g* (Allegra-64R, Beckman Coulter, USA) at 4 °C for 30 min and 10,000 *g* at 4 °C for 1 h. Exosomes in the supernatant were subsequently pelleted by ultracentrifugation (Beckman XPN-100, Beckman, USA) at 150,000 *g* at 4 °C for 2 h, and the pellet was suspended in phosphate-buffered saline (PBS). The supernatant was then filtered with a 0.22-μm filter and the final exosome suspension was stored at −80 °C until use. The morphology of GExos was observed by transmission electron microscopy (TEM) (Talos L120C, Thermo Fisher Scientific, USA). The total exosome protein concentrations were determined by using the BCA Protein Assay kit (Boster Biological Technology Co. Ltd., Wuhan, China). The particle sizes and surface charge (represented by the surface zeta potential) were measured using laser diffraction spectrometry (Malvern Zeta sizer 3000HS, Malvern) (Brookhaven Instruments Corp, Holtsville, NY, USA).

### GExos miRNAs, lipids, small molecules, and protein isolation and analysis

The analysis of G-E-miRNAs was described previously [28]. In brief, the total RNA of GExos was isolated and purified using Trizol reagent (Invitrogen, Carlsbad, CA, USA), then fragmented into small pieces under high temperature. The cleaved RNA fragments were reverse-transcribed to create the cDNA, which was next used to synthesize U-labeled second-stranded DNAs. After the heat-labile UDG enzyme treatment of the U-labeled second-stranded DNAs, the ligated products are amplified with PCR. The average insert size for the final cDNA library was 300 bp (±50 bp). Last, we performed the paired-end sequencing on an Illumina Hiseq 4000 (LC Bio, China) following the vendor’s recommended protocol. For GExos miRNA analysis, sequences with a length of 18 to 25 nucleotides were mapped to specific species precursors in miRBase 22.0 by BLAST search.

Lipids of GExos were extracted according to the MTBE method. Briefly, samples (100 μl) were spiked with internal lipid standards, then homogenized with 100 μl of water and 240 μl of methanol. After that, 800 μl of MTBE was added and the mixture was treated with ultrasound for 20 min at 4 °C followed by sitting still for 30 min at room temperature. The solution was centrifuged at 14,000 *g* for 15 min at 10 °C and the upper layer was obtained and dried under nitrogen. The lipid extracts were re-dissolved in 200 μl of 90% isopropanol/acetonitrile, centrifuged at 14,000 *g* for 15 min, and finally 3 μl of the sample was injected. The reverse-phase chromatography was selected for LC separation using the CSH C18 column (1.7 μm, 2.1 mm × 100 mm, Waters). Electrospray ionization (ESI) was used to detect positive and negative ions. Analyses were performed using a UHPLC Nexera LC-30A ultra-performance liquid chromatography system (SHIMADZU, Japan) coupled to Q-Exactive Plus (Thermo Fisher Scientific) in Shanghai Applied Protein Technology Co., Ltd. Finally, Lipidsearch (Thermo Fisher Scientific, USA) was used to extract and identify the peaks of lipid molecules and internal standard lipid molecules. The main parameters were as follows: precursor tolerance: 5 parts per million (ppm), product tolerance: 5 ppm, and production threshold: 5%.

The small molecules contained in GExos were isolated by the HPLC method. In brief, 400 μl of GExos and 200 μl of radio immunoprecipitation assay (RIPA) lysate were mixed and ultrasonicated 3 times at 450 W, 5 min each time in an ice bath. Then, the extract was centrifuged at 13,000*g* for 5 min at 4 °C. The supernatant fractions were separated using HPLC. The reverse-phase chromatography was selected for LC separation using the CSH C18 column (5 μm, 4.6 mm × 250 mm, carbon loading: 11%). Mobile phase A is acetonitrile and mobile phase B is 0.1% phosphoric acid solution. The small molecules were eluted at 1.3 ml/min using an acetonitrile gradient consisting of 81% B for 30 min, 81% to 76% B over 5 min, and 76% to 60% B over 25 min before returning to 81% B over 0.5 min. Column temperature was 30 °C and detection wavelength was 203 nm. Rg_1_, Rb_1,_ and Re standards were dissolved in methanol, and the content of GExos was quantified using an external standard method.

GExos-containing proteins were isolated with SDT (4% SDS and 100 mM Tris-HCl, pH 7.6) buffer for lysis and extraction. The amount of protein was quantified with the BCA Protein Assay Kit (Bio-Rad, USA). Twenty micrograms of protein for each sample was mixed with 5× loading buffer respectively and boiled for 5 min. The proteins were separated on 4% to 20% sodium dodecyl sulfate-polyacrylamide gel electrophoresis (SDS-PAGE) gel (constant voltage 180 V, 45 min). Protein bands were visualized by Coomassie Blue R-250 staining.

### Cell lines and cell culture

Human umbilical vein endothelial cells (HUVECs) were obtained from the cell bank of the Chinese Academy of Sciences (Shanghai, China). The HUVECs were cultured in RPMI 1640 medium containing 10% fetal bovine serum (Gibco BRL), L-glutamine, penicillin (50 U/ml), and streptomycin (50 U/ml). Cells were maintained at 37 °C with 5% CO_2_.

### Cellular uptake of GExos by HUVECs

For confocal laser scanning microscopy imaging, GExos were labeled with CM-Dil (Yeasen, China) according to the manufacturer’s instructions. The unbonded dye was depleted by ultracentrifugation at 150,000*g* for 2 h at 4 °C. HUVECs were incubated with labeled exosomes (8 μg/ml) at 37 °C. After being fixed by 4% paraformaldehyde, actin was stained with phalloidin and the nuclei were stained with 4′,6-diamidino-2-phenylindole (DAPI, Invitrogen, USA), followed by observation using confocal laser scanning microscopy (IX81-FV1000, Olympus, Japan). For flow cytometry analysis, GExos were labeled with CM-Dil, and the labeled GExos were then ultracentrifuged at 150,000*g* for 2 h to remove the free dye and added to HUVECs. Cells were then counted at each time point (0, 3, 6, 12, or 24 h) via flow cytometry (CytoFlex S, Beckman, USA). Intact cells were selected by size gating for analysis of fluorescence staining. Data were analyzed by FlowJo software.

### HUVEC proliferation assay

For cell proliferation analysis, HUVECs were plated in supplemented RPMI 1640 medium and allowed to attach overnight. The next day (time 0), the medium was replaced with supplemented RPMI 1640 with or without HG or GExos treatments for 24 h. Cell proliferation was then evaluated by the Cell Counting Kit-8 assay (Beyotime) according to the manufacturer’s instructions. Briefly, 10 μl of CCK-8 was added to each well (100 μl of culture medium). After incubation for 2 h at 37 °C, the absorbance at 450 nm was measured by BioTek Cytation^3^ CellImaging Multi-Mode Reader.

### HUVEC scratch assay for cell migration

HUVECs were plated onto 24-well cell culture plates at a density of 80,000 cells per well in supplemented RPMI 1640 medium and allowed to attach overnight. Monolayers were then wounded using the same 200-μl pipette tip. The medium was then replaced with supplemented RPMI 1640 (without serum) with or without HG or drug treatments and images of wounds were taken every 6 h until closure.

### HUVEC tube formation assay

Tube formation assay was performed according to a published protocol. Eighty microliters of GFR Matrigel (Corning, USA) was coated onto each well of a 48-well plate. Then, the plate was incubated at 37 °C for 30 min. Next, HUVECs (30,000 cells) were seeded into each well of the plate, and the medium was replaced with supplemented RPMI 1640 with or without HG or GExos treatments for 3 h, 6 h, and 9 h. Images were taken with a microscope (ECLIPSE-TI-S, Nikon, Japan). The obtained images were quantitatively analyzed by the Angiogenesis Analyzer plugin and ImageJ to determine the tube length and the number of junctions. Each experiment was performed in triplicate.

### RNA-seq data generation and analysis

Total RNA was extracted using TRIzol (Thermo Fisher Scientific, 15596018) according to the manufacturer’s instructions including a DNase treatment. RNA concentration and purity were determined spectrophotometrically using the NanoDrop ND-1000 (NanoDrop, Wilmington, DE, USA) and RNA integrity was assessed using a Bioanalyzer 2100 (Agilent, CA, USA). Using the Illumina paired-end RNA-seq approach, we sequenced the transcriptome, generating a total of million 2 × 150 bp paired-end reads. Prior to mapping, the low-quality reads (1, reads containing sequencing adaptors; 2, reads containing sequencing primer; 3, nucleotide with a quality score lower than 20) were removed. After that, about 8 G bp of each sample of cleaned, paired-end reads were produced. We next aligned reads of treated cells and blank cells to the reference genome using HISAT2. The mapped reads of each sample were assembled using StringTie. Then, all transcriptomes from samples were merged to reconstruct a comprehensive transcriptome using perl scripts. After the final transcriptome was generated, StringTie and edgeR were used to estimate the expression levels of all transcripts. StringTie was used to perform expression levels for mRNAs by calculating FPKM. Gene set enrichment analysis (GSEA) was performed using the Broad Institute GSEA v4.2.3 software[29,30] (http://www.gsea-msigdb.org/gsea/index.jsp).

### RNA extraction and real-time PCR analysis

Cells or tissues were collected and the total RNAs were extracted using the TRIzol Plus RNA Purification Kit (Thermo Fisher Scientific, Carlsbad, CA, USA) as described by the manufacturer’s protocol. cDNA was synthesized using the SuperScript III First-Strand Synthesis SuperMix for quantitative real-time polymerase chain reaction (qRT-PCR) (Thermo Fisher Scientific, 11752-050, Carlsbad, CA, USA) following the manufacturer’s instructions. Real-time PCR was performed with PowerUp SYBR Green Master Mix (Applied Biosystems, 4367659, Carlsbad, CA, USA) according to the manufacturer’s guidelines. The cycling program used was 95 °C for 2 min, followed by 40 cycles of amplification (95 °C for 15 s, 60 °C for 1 min). Relative expressions of target genes were normalized to GAPDH, evaluated by the 2^−∆∆Ct^ method, and given as a ratio to control in the experiment.

### Western blotting analysis

Proteins from cells or tissues were extracted with RIPA buffer (Thermo Fisher Scientific, 89900, USA) supplemented with Protease and Phosphatase Inhibitor Cocktail (Thermo Fisher Scientific, 87785, USA), and then quantified using the BCA protein assay kit (Beyotime Biotechnology, P0010, China). About 50 μg of protein was loaded onto each lane of SDS-PAGE gel and transferred to a Hybond-P polyvinylidene fluoride membrane (IPVH00010, Millipore, USA). After blocking with 5% non-fat milk in Tris-buffered saline containing 0.1% Tween-20, the membranes were then incubated at 4 °C overnight with the following antibodies: rabbit anti-PFKM (Abcam, ab154804, 1:1,000), rabbit anti-PGK1 (Abcam, ab199438, 1:2,000), rabbit anti-ENO1 (Abcam, ab227978, 1:1,000), rabbit anti-PGLS (Abcam, ab229980, 1:2,000), rabbit anti-ACACA (Abcam, ab45174, 1:2,000), rabbit anti-PDHA1 (Abcam, ab168379, 1:2,000), rabbit anti-β-actin (Abcam, ab68477, 1:10,000, internal control), rabbit anti-Cyclin D1 (Abcam, ab134175, 1:10,000), mouse anti-Cyclin D3 (Abcam, ab289546, 1:1,000), and mouse anti-β-actin (Abcam, ab8226, 1:1,000, internal control). Protein expression was visualized on x-ray films using horseradish peroxidase-labeled goat anti-rabbit secondary antibody (1:5,000, Thermo Fisher Scientific, 31210, USA) or goat anti-mouse secondary antibody (1:5,000, Thermo Fisher Scientific, 31431, USA) by SuperSignal West Dura Extended Duration Substrate (Thermo Fisher Scientific, USA). Band intensities were quantitated using ImageJ 1.8.1 software. Data are presented as the ratio of the optical density of the target protein band to the β-actin band.

### Metabolites measurement

HUVECs were plated in supplemented RPMI 1640 medium and allowed to attach overnight. The medium was then replaced with supplemented RPMI 1640 medium with or without HG or GExos treated for 24 h. Cells were collected, and pellets were flash-frozen in liquid nitrogen in cryovials. Metabolite extraction and sample analysis: Cell samples, glass bead, and acetonitrile (ACN):methanol:H_2_O mixed solution (2:2:1, v/v/v) were added into a centrifuge tube and subjected to 3 freeze–thaw cycles alternating between liquid nitrogen and a 37 °C water bath. Next, samples were centrifuged at 12,000*g* for 10 min at 4 °C, and the supernatant was concentrated and dried. Then, 300 μl of acetonitrile:2-amino-3-(2-chloro-phenyl)-propionic acid (4 ppm) solution prepared with 0.1% formic acid (1:9, v/v) was accurately added to redissolve the sample, the supernatant was filtered by a 0.22-μm membrane, and the mixture was transferred into a detection bottle for LC-MS detection.

Liquid chromatography conditions: The LC analysis was performed on a Vanquish UHPLC System (Thermo Fisher Scientific, USA). Chromatography was carried out with an ACQUITY UPLC HSS T3 (150×2.1 mm, 1.8 μm) (Waters, Milford, MA, USA). The column was maintained at 40 °C. The flow rate and injection volume were set at 0.25 ml/min and 2 μl, respectively. For LC-ESI (+)-MS analysis, the mobile phases consisted of (C) 0.1% formic acid in acetonitrile (v/v) and (D) 0.1% formic acid in water (v/v). Separation was conducted under the following gradient: 0 to 1 min, 2% C; 1 to 9 min, 2% to 50% C; 9 to 12 min, 50% to 98% C; 12 to 13.5 min, 98% C; 13.5 to 14 min, 98% to 2% C; 14 to 20 min, 2% C. For LC-ESI (−)-MS analysis, the analytes were carried out with (A) acetonitrile and (B) ammonium formate (5 mM). Separation was conducted under the following gradient: 0 to 1 min, 2% A; 1 to 9 min, 2% to 50%A; 9 to 12 min, 50% to 98% A; 12 to 13.5 min, 98% A; 13.5 to 14 min, 98% to 2% A; 14 to 17 min, 2% A.

Mass spectrum conditions: Mass spectrometric detection of metabolites was performed on Orbitrap Exploris 120 (Thermo Fisher Scientific, USA) with ESI ion source. Simultaneous MS1 and MS/MS (full MS-ddMS2 mode, data-dependent MS/MS) acquisition was used. The parameters were as follows: sheath gas pressure, 30 arb; aux gas flow, 10 arb; spray voltage, 3.50 kV and −2.50 kV for ESI (+) and ESI (−), respectively; capillary temperature, 325 °C; MS1 range, m/z 100 to 1,000; MS1 resolving power, 60,000 full width half maximum; the number of data-dependent scans per cycle, 4; MS/MS resolving power, 15,000 full width half maximum; normalized collision energy, 30%; dynamic exclusion time, automatic.

### Metabolomic metabolite analysis

The metabolites were identified by accuracy mass (< 30 ppm) and MS/MS data, which were matched with HMDB (http://www.hmdb.ca), mass bank (http://www.massbank.jp/), LipidMaps (http://www.lipidmaps.org), McCloud (https://www.mzcloud.org), and KEGG (http://www.genome.jp/kegg/). Finally, *P* value<0.05 and VIP values>1 were considered to be statistically significant metabolites. Differential metabolites were subjected to pathway analysis by MetaboAnalyst (https://www.metaboanalyst.ca/), which combines results from powerful pathway enrichment analysis with the pathway topology analysis. The identified metabolites in metabolomics were then mapped to the KEGG pathway for biological interpretation of higher-level systemic functions. The gene–metabolite joint analysis was performed by MetaboAnalyst.

### Glycolytic metabolite analysis

HUVECs were plated in supplemented RPMI 1640 medium and allowed to attach overnight. The next day (time 0), the medium was replaced with supplemented RPMI 1640 with or without HG or GExos treatments for 24 h. In total, 3 cellular metabolite assay kits were used to detect the content of intracellular glycolytic metabolites. The glucose-6-phosphate assay kit was purchased from Beyotime (S0185, Shanghai, China), the fructose-1,6-diphosphate assay kit was purchased from Solarbio Life Science (BC2240, Beijing, China), and the Acetyl Co-A Activity Assay Kit was purchased from Solarbio Life Science (BC0980, Beijing, China). All experiments followed the manufacturer’s protocol.

### ROS and eNOS staining

In vitro, HUVECs were plated in supplemented RPMI 1640 medium and allowed to attach overnight. The next day (time 0), the medium was replaced with supplemented RPMI 1640 with or without HG or GExos treatments for 24 h. In vivo, the tissues of the wound regions were retrieved on day 8 after the treatment and embedded in an optimal cutting temperature (OCT) compound, followed by freezing and slicing into 15-μm-thick sections at −22 °C. Intracellular ROS level was assayed by the fluorescent probe dichloro-dihydro-fluorescein diacetate (DCFH-DA) (Beyotime, S0033S, Shanghai, China). In brief, cells were incubated with 10 μmol/l DCFH-DA at 37 °C for 20 min in the dark. Then, the cells were washed twice using cold PBS. The fluorescence images of intracellular ROS were acquired by using fluorescence microscopy (OLYMPUS BX61). The eNOS expression levels were evaluated by eNOS stain kit (Solarbio Life Science, G3400, Beijing, China) following the manufacturer’s protocol. In brief, reduced coenzyme II (NADPH) is the coenzyme of nitric oxide synthase, which can dehydrogenate the substrate, and then transfer the hydrogen to nitrotetrazolium blue (NBT). NBT will be reduced to a blue–black precipitate, and the precipitate site is the site of NADPH, namely, the eNOS site.

### Measurement of intracellular ATP, extracellular glucose, and nitric oxide

HUVECs were plated in supplemented RPMI 1640 medium and allowed to attach overnight. The next day (time 0), the medium was replaced with supplemented RPMI 1640 with or without HG or GExos treatments for 24 h. The cells were used for intracellular adenosine triphosphate (ATP) detection while the cell supernatant was used for extracellular glucose and NO detection. The concentration of ATP and glucose was evaluated by ATP detection assay kit (Beyotime, S0026, Shanghai, China) and glucose detection assay kit (Beyotime, S0201S, Shanghai, China), following the manufacturer’s protocol, respectively. The NO level was determined by the NO detection assay kit (Elabscience, E-BC-K035-M, Wuhan, China). In brief, NO is easily oxidized to form NO^2-^ in vivo or in aqueous solution, and a reddish azo compound is formed with the color developing agent, and the concentration of the azo compound is linearly related to the concentration of NO. The concentration of NO can be calculated indirectly by measuring the OD value at 550 nm.

### Animal study

In total, 8- to 12-week-old male B6.BKS(D)-*Lepr ^db^*/J (*db/db*) mice were used in the studies. All mice were purchased from Shanghai SLAC Laboratory Animal Co. Ltd. and were maintained in mouse barrier facilities. All animal experimental procedures were performed in obedience to the guidelines and protocols of the Animal Experimental Ethics Committee of Zhejiang University (ZJU20170733). Full-thickness wounds were made as previously described. In brief, wounds with a diameter of 12 mm were made on shaved back skin by scalpel excision under analgesia and general anesthesia in 8- to 12-week-old male B6.BKS(D)-*Lepr^db^*/J (*db/db*). A donut-shaped silicone splint with a 15-mm diameter was centered around the wound and affixed to the skin using adhesive and interrupted nylon sutures. For treatment, mice with skin wounds were randomly divided into 3 experimental groups (*n* = 8 mice per group). The wounds were treated with either 100 μl of PBS or 100 μg/ml or 200 μg/ml GExos suspension, respectively, for the direct contact with the topical ECs. The ulcer sites were then stuck with a sterile dressing to ensure the close attachment of GExos to the topical site, which was replaced every other day, at which time digital photographs of the wounds were taken until wounds were fully reepithelialized. Wound areas were digitally measured relative to the inner area of silicone rings, using Adobe Photoshop CS6 (San Jose, CA), and normalized to original wound areas. The weight of all mice was also recorded. The blood glucose of the mice and wound sites were measured by a glucometer (Sinocare, China) and recorded.

### Immunofluorescence tissue staining

The tissues of the wound regions were retrieved on days 8 and 16 after the treatment and embedded in an optimal cutting temperature compound, followed by freezing and slicing into 10-μm-thick sections at −22 °C. To visualize the regenerated angiogenesis in vivo, tissue sections were stained with CD31. CD31 signals were visualized using the fluorescein isothiocyanate and Cy3-conjugated secondary antibodies, respectively. All nuclei were stained by DAPI. Images of the samples were observed under confocal laser scanning microscopy (VS120, Olympus). To quantify the fluorescence intensity, images were photographed from 4 random areas. All images were post-processed and quantified using ImageJ software.

### Microvascular imaging and analysis

Microvascular imaging and analysis were performed for the healed skin tissues, 16 days after skin wound treatment. In brief, the different groups of fresh skin tissue around the wound were extracted and flattened to expose the dermis of the skin upwards. The photos of microvessels in the newborn area were observed under a body-type microscope (HDCE-X5N, China). All images were post-processed and quantified using ImageJ software.

### Histology analysis

Histological analysis was performed for the healed skin tissues and organs, 8 days and 16 days after skin wound treatment. Retrieved samples were fixed in 4% buffered paraformaldehyde, dehydrated, and then embedded in paraffin or OCT compound for slice preparation. The slice sections (5 μm thick) were stained with hematoxylin and eosin (H&E; Keygen Biotech), according to the manufacturer’s protocol. The stained skin sections were observed and photographed with confocal laser scanning microscopy. Additionally, the organs were extracted. The heart, liver, spleen, lung, and kidney were cut into smaller sections, fixed in 4% paraformaldehyde, embedded in paraffin, and sectioned into 5-μm slices. The organ sections were stained with H&E and were visualized by confocal laser scanning microscopy for the histological study of toxicity.

### Statistical analysis

Unless otherwise stated, data were expressed as mean ± standard deviation. For comparisons between 2 groups, means were compared using unpaired 2-tailed Student’s *t* tests. A one-way analysis of variance with post hoc Tukey’s honest significant difference was conducted for multiple sample analyses. All statistical analyses were performed using GraphPad Prism version 9.1.1 software (GraphPad Software Inc.).

## Results

### Preparation and characterization of GExos

**Fig. 2.**
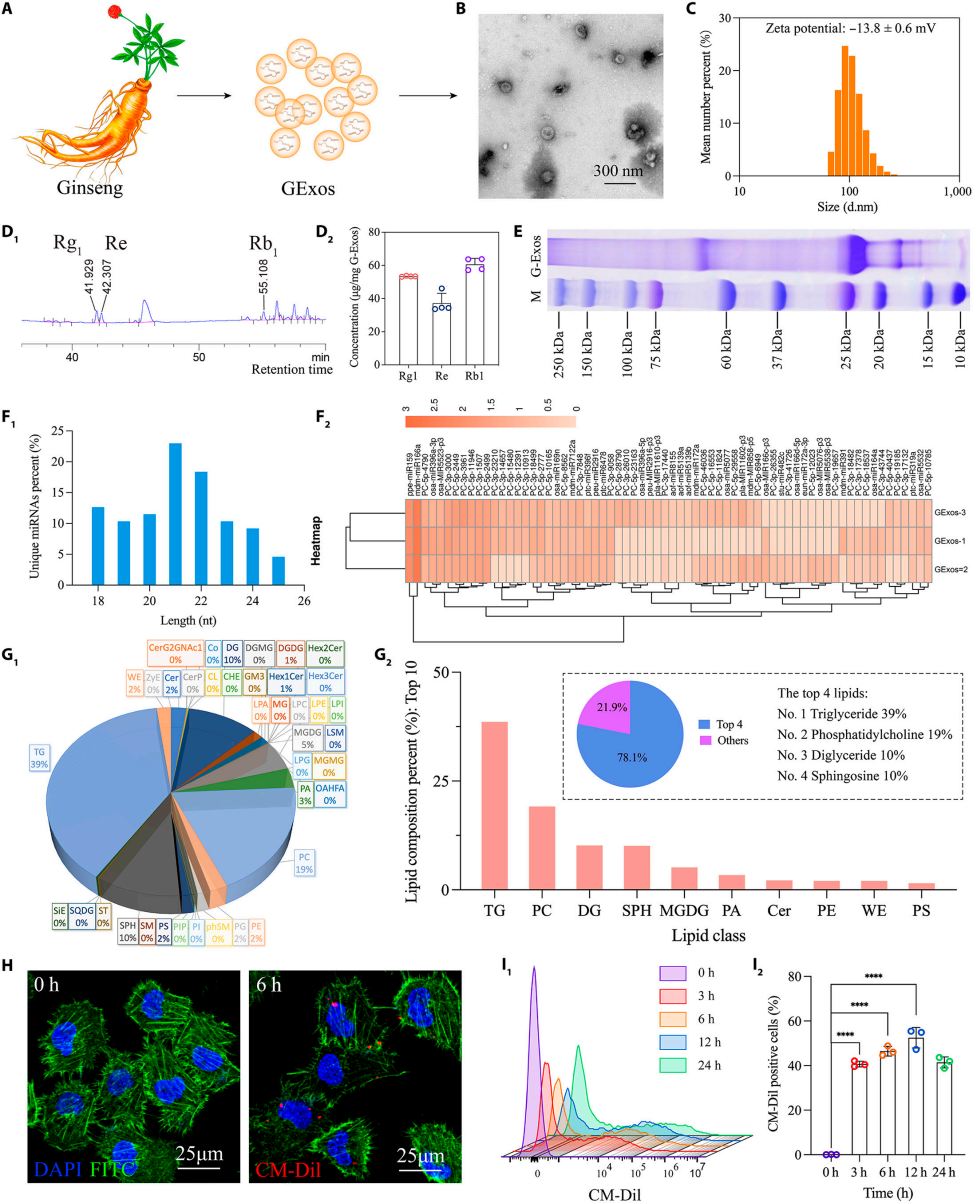
Preparation, characterization, composition analysis, and cellular uptake of GExos in ECs. (A) Schematic fabrication of the GExos. (B) SEM image of GExos. (C) Size distribution and zeta potential of GExos. HPLC fingerprints of small molecules (D_1_) and quantitative analysis of Rg_1_, Re, and Rb_1_ (D_2_) in GExos. (E) Isolation and Coomassie bright blue staining of proteins in GExos. Length distribution of miRNAs (F_1_) and heatmap of the expressed miRNAs (F_2_) in GExos. (G_1_) Relative quantification analysis of lipid classes in GExos. (G_2_) Normalized expression values and the total proportion of top 4 lipids in GExos. (H) Cellular uptake of CM-Dil (red) labeled GExos into HUVECs. The nuclei and the cytoskeleton were stained with DAPI (blue) and F-actin (green), respectively. (I_1_ and I_2_) CM-Dil-positive rates of HUVECs co-cultured with the CM-Dil-labeled GExos for 0, 3, 6, 12, and 24 h, analyzed by flow cytometry.

GExos were isolated and purified from ginseng, which do not contain toxic or disease-related molecules (Table S1), by our previously reported protocol [28] (Fig. 2A). GExos were extracted using a standardized process every time, and the extraction efficiency of GExos was quantified by measuring particle numbers and protein content. The results showed that about 4.83×10^10^ GExos particles could be obtained from 50 g of fresh plant ginseng, and the corresponding protein amount was 12 mg (Fig. S1). Given that the active ingredients contained in GExos are the material basis for its therapeutic effects, the quantity of GExos in the succeeding studies was quantitatively expressed by measuring the concentration of protein in the GExos suspension. As shown, the prepared GExos were shown for the characteristic cup shape under TEM observation and distributed well without aggregation (Fig. 2B). The particle size of GExos was determined to be 117.7 ± 6.3 nm (Fig. 2C), and GExos of this size have a zeta potential of −13.8 ± 0.6 mV. Our recent study demonstrated that plant-derived exosomes could be stably stored at −80 °C when they are in liquid form [18], and their particle size and morphology did not change significantly even after 1 year of storage. Therefore, the newly extracted GExos are stored at −80 °C in liquid form. Before use, the GExos suspension was gradually thawed to room temperature for experimentation.

Exosomes possess a remarkable loading capacity for the endogenous substances from the mother cells [31,32]; thus, we characterized the incorporated components in GExos. Several widely reported ginsenosides Rg_1_, Re, and Rb_1_ [33] were determined, and their concentrations in GExos were 53.39 ± 0.27 μg/mg, 37.16 ± 5.98 μg/mg, and 60.62 ± 3.62 μg/mg, respectively (Fig. 2D_1_ and D_2_). The gel electrophoresis result shows that the molecular weight of most proteins contained in GExos was distributed around 20 to 25 and 65 kDa (Fig. 2E). Nucleic acids, including DNA, mRNA, and miRNA, are major components contained in GExos, which were also extracted and analyzed (Fig. S2). Among these, DNA requires transfer to the nucleus to exert its function, while mRNA is known for its instability and susceptibility to degradation. In contrast, miRNAs are highly conservative nucleic acids with bioactivities and stable characteristics, making them one of the most important bioactive contents in exosomes [34]. In this study, 87 miRNAs with a length distribution of 18 to 25 nt (Fig. 2F_1_) were detected in GExos (Fig. 2F_2_). These miRNAs show immense potential in modulating angiogenesis processes and behaviors (Fig. S3).

The compositional structure of exosomal lipid bilayers has garnered significant attention in the field of drug delivery [35]. Figure 2G_1_ shows the lipid compositions of GExos (Table S2). The top 4 classes of lipids in the GExos membrane contribute 78.1% of the total lipids (Fig. 2G_2_), including triglyceride (39%), phosphatidylcholine (19%), diglyceride (10%), and sphingosine (10%). Among these, phosphatidylcholine is one of the most abundant phospholipids contained in most mammalian cell membranes [36] that may act as significant promoters to the cellular uptake and transmembrane of exosomes to mammalian cells by lipid fusion [37]. Sphingosine forms a primary part of sphingolipids, which is a class of cell membrane lipids, whose metabolites are lipid signaling molecules involved in endocytosis and regulating the actin cytoskeleton [38]. These components indicate the obvious efficacy of GExos in cellular uptake by mammalian cells, permitting the transfer of the encapsulated substances to target cells for therapeutic regulation.

We further investigated the cellular uptake and influence of GExos in a HUVEC model to evaluate its potential to stimulate angiogenesis. A time-dependent internalization of GExos in HUVECs (Fig. 2H and Fig. S4), with the increasing CM-Dil^+^ HUVECs reaching 39.8%, 49.4%, and 55.5% at 3 h, 6 h, and 12 h, respectively, was received (Fig. 2I_1_ and I_2_ and Fig. S5). This remarkable and swift uptake of GExos by HUVECs might be attributed to the elevated phosphatidylcholine and sphingosine contents in the membrane of GExos (Fig. 2G), which facilitate not only the lipid fusion, but also lipid raft-, caveolin-, or clathrin-mediated phagocytosis, micropinocytosis, and endocytosis of GExos in HUVECs.

Cellular uptake mediates the transfer of incorporated substances, especially the small nucleic acids, between exosomes and target cells [39], which led us to investigate whether the endogenous miRNAs can be transferred into HUVECs by GExos. For which, the miRNAs contained in GExos are remapped to the human gene bank for annotation (Figs. S6 and S7). Then, the RNA-seq analysis revealed that 25 pivotal miRNAs were transported into HUVECs by GExos (Fig. S8), which may further contribute to the functions of extracellular matrix organization and vasculogenesis (Fig. S9). Remarkably, the crucial roles in angiogenesis of a significant subset of these identified miRNAs have been reported, including notable representatives such as the let-7 family and the miR-17-92 cluster [40] (Table S3).

### Influence of GExos in the angiogenesis of HUVECs under HG conditions

**Fig. 3.**
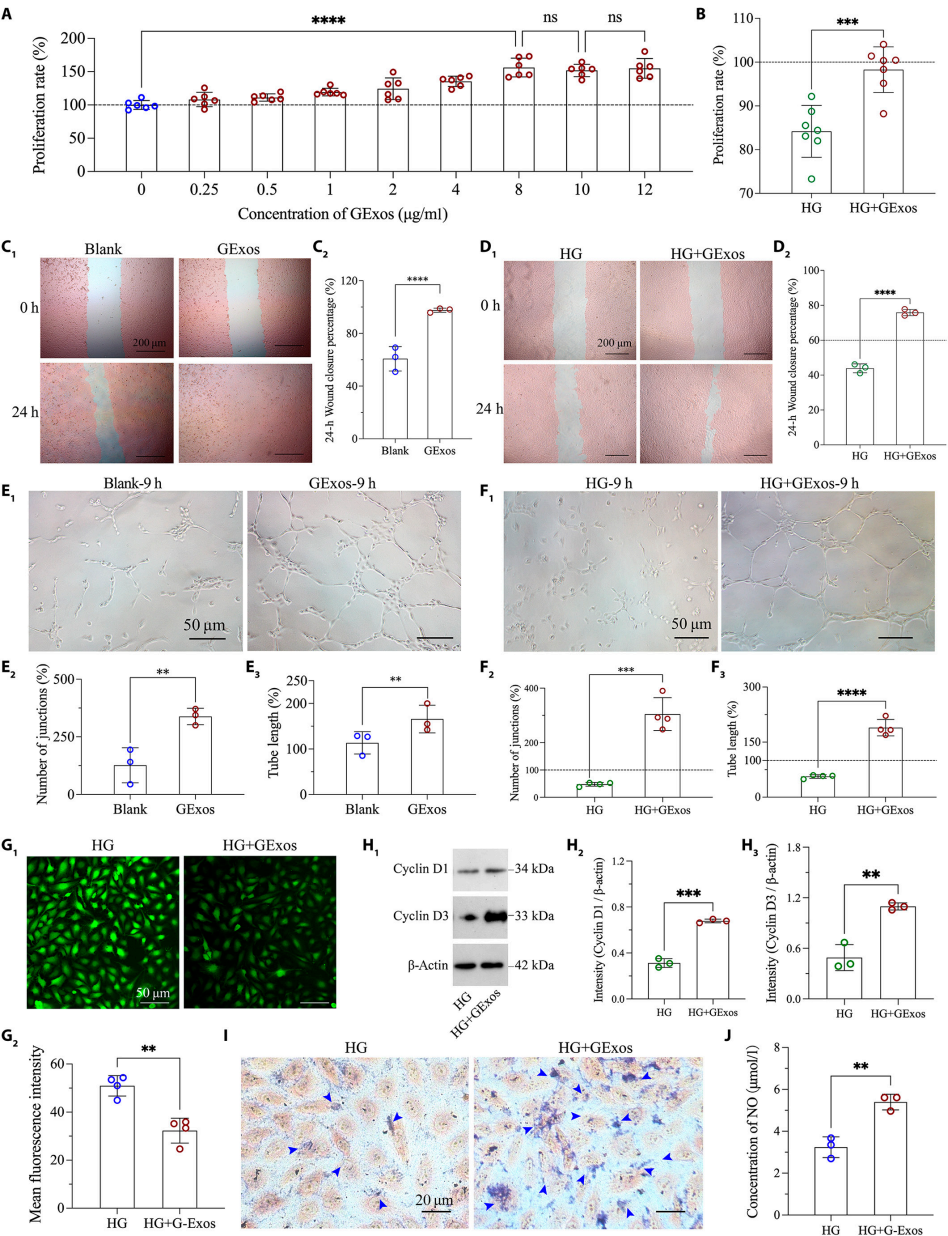
GExos enhance proliferation, migration, and tubule formation of HUVECs in HG culture. (A) HUVECs’ viability against GExos treatment. (B) The proliferation rates of HUVECs treated with HG or HG+GExos at 24 h after co-incubation. Light microscope images and quantified migration of HUVECs in blank or GExos (C_1_ and C_2_) and HG or HG+GExos (D_1_ and D_2_) groups, respectively. Light microscope images and quantified tubule formation of HUVECs on matrigel in blank or GExos (E_1_ to E_3_) and HG or HG+GExos (F_1_ to F_3_) groups, respectively. All black lines in the above figures indicate the level of blank group. The ROS fluorescence staining (G_1_) and quantitative analysis (G_2_) of HUVECs in the HG or HG+GExos group. Expression levels (H_1_) and quantitative analysis (H_2_ and H_3_) of Cyclin D1 and Cyclin D3 in the HG or HG+GExos group. The eNOS staining (I) and quantitative analysis of NO secretion (J) of HUVECs in the HG or HG+GExos group. *P* values are shown: *P < 0.05, **P < 0.01, ***P < 0.001, ****P < 0.0001.

The HG environment often leads to the dysfunction of ECs, which is a primary factor contributing to inadequate angiogenesis. HUVECs have been widely used as the representative EC model for various angiogenesis studies because of their advantages of easy access, high stability, wide application, and strong plasticity.[41–43] To identify the influence of GExos in angiogenesis, we evaluated and compared the angiogenesis in 4 tested groups: HUVECs cultured under normal glucose conditions (Blank), HUVECs cultured under normal glucose conditions with GExos treatment (GExos), HUVECs cultured under HG conditions, and HUVECs cultured under HG conditions with GExos treatment (HG+GExos). Figure 3A shows that the proliferation rates of HUVECs were significantly enhanced with the increase of GExos concentration (from 0 to 12 μg/ml), and an optimal stimulating concentration was obtained at 8 μg/ml, which was used in all subsequent in vitro experiments. As expected, the proliferation efficacy of HUVECs in the HG group is markedly reduced; however, the HG+GExos group expressed a 1.2-fold higher proliferative efficiency than that of the HG group, and nearly the same as that of the blank group, indicating that GExos could reverse the impaired proliferation of HUVECs by HG (Fig. 3B). Cell migration experiment further verified the angiogenic effect of GExos, with the highest scratching closure of nearly 100% at 24 h post-treatment received. By contrast, just a 60.71% closure rate was received in the blank group (Fig. 3C). The migration ability of HUVECs was impaired by HG with a limited closure rate of only 43.88%, while a surprising result of 75.87% was obtained in the HG+GExos group (Fig. 3D). Similar results were also observed in tubule formation tests; the number of junctions and tube length of the GExos group were significantly increased, which were 2.00- and 1.44-fold higher than those of the blank group at 9 h post-treatment, respectively (Fig. 3E_1_ to E_3_). In the HG+GExos group, a drastic increased number and length of endothelial junctions and tubes was received, which was around 2.5-fold higher than the damaged efficacy of the HG group (Fig. 3F_1_ to F_3_). All these results provide compelling evidence that GExos exhibit profound angiogenic effects, capable not only of promoting the proliferation, migration, and tubule formation of HUVECs, but also of further reversing HG-induced endothelial dysfunction and stimulating angiogenesis.

Many studies have revealed that HG-induced reactive oxygen species (ROS) accumulation in HUVECs is the key factor for impaired angiogenesis [1,2]. Excessive ROS act on the Cyclin-dependent kinases to inhibit Cyclin D_1_ and Cyclin D_3_ protein expression, as well as lead to the lack of eNOS/NO to impair angiogenesis [4]. Thus, we detected the expression changes of these representative molecules in HG culture with GExos treated or not. As expected, obvious ROS expression in HUVECs was observed in the HG group (Fig. 3G_1_), which was 1.58-fold higher than that of the HG+GExos group (Fig. 3G_2_). Meanwhile, the enhanced expression of Cyclin D_1_ and Cyclin D_3_ proteins was detected in HG+GExos-treated HUVECs, which was nearly 2-fold higher than that of the HG group (Fig. 3H_1_ to H_3_). Furthermore, a large amount of eNOS expression was shown in the HG+GExos group (Fig. 3I), and the NO secretion was up-regulated in the HG+GExos group, which was 1.67-fold higher than that of the HG group (Fig. 3J). These results give definite evidence in that GExos have unique features in reversing the HG-induced endothelial dysfunction partly by down-regulating the ROS-induced pathological activities in ECs.

### HUVEC genetic and metabolic alteration to GExos treatment in HG culture

The reversal of endothelial dysfunction ability and the excellent pro-angiogenic efficacy of GExos in HG culture (Fig. 3) prompted further molecular mechanism studies. Studies in the last decade have emphasized the crucial role of glycolysis in angiogenesis and HG-induced endothelial dysfunction [44]. The glycolysis comprises 2 major steps of anaerobic glycolysis and oxidative phosphorylation, where the activation of anaerobic glycolysis contributes to glucose rapid transformation and energy rapid generation to stimulate angiogenesis, while the up-regulation of oxidative phosphorylation in HG environment is one of the major pathways for ECs’ ROS production [45].

**Fig. 4.**
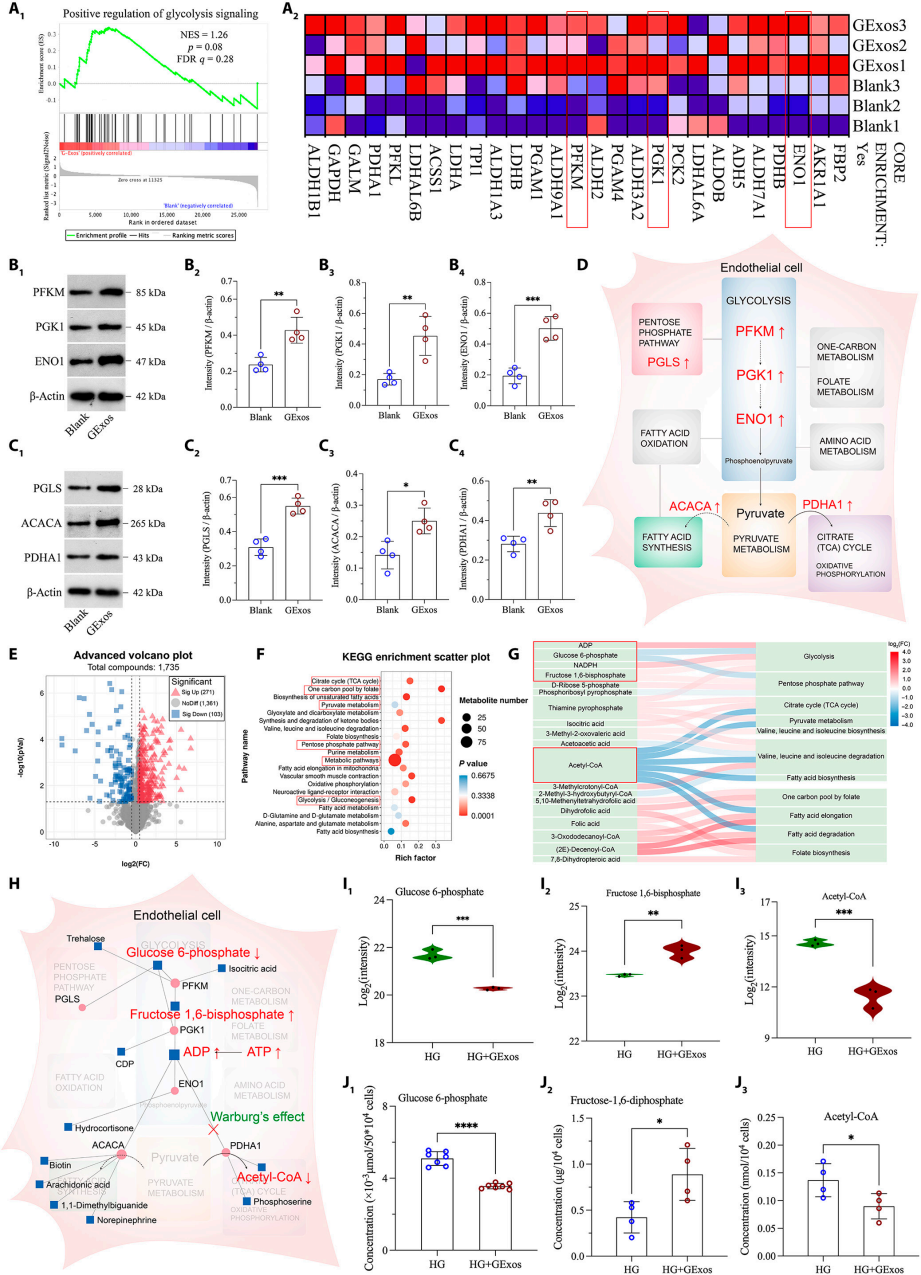
HUVECs’ genetic and metabolic alterations to GExos treatment in HG culture. (A1 to A2) Enrichment plots from GSEAs of gene sets for the “Positive regulation of glycolysis signaling”. Expression levels and quantitative analysis of (B1 to B4) PFKM, PGK1, and ENO1, and (C1 to C4) PGLS, ACACA, and PDHA1. (D) Schematic illustration of the major genes and metabolic signaling pathways relevant to angiogenesis, regulated by GExos. (E) Volcano plots of differentially expressed metabolites identified at *q* < 0.05. (F) KEGG pathway enrichment analyses of the differentially expressed metabolites. (G) Sankey diagram of the differentially expressed metabolites and corresponding enrichment pathways. (H) Mechanism diagram of gene–metabolite interaction. Expression levels (I_1_ to I_3_) and quantitative analysis (J_1_ to J_3_) of G6P, FDP, and Acetyl-CoA. *P* values are shown: *P < 0.05, **P < 0.01, ***P < 0.001, ****P < 0.0001.

The GSEA revealed the up-regulation of glycolysis signaling in HUVECs by GExos treatment (Fig. S10 and Fig. 4A). Consequently, pivotal differentially expressed genes were scrutinized, including PFKM, PGK1, and ENO1, which respectively function as rate-limiting enzymes in the initial, intermediate, and final stages of anaerobic glycolysis [46–48]. Subsequent RT-qPCR results revealed a significant up-regulation of PFKM, PGK1, and ENO1 genetic levels upon GExos treatment (Fig. S11), and the expression of PFKM, PGK1, and ENO1 proteins also increased by 1.81-fold, 2.66-fold, and 2.59-fold, respectively (Fig. 4B_1_ to B_4_), indicating that the enhanced anaerobic glycolytic signaling by up-regulating these genes potentially drives GExos’ capacity to stimulate ECs proliferation, migration, and tubule formation [44,45]. Notably, the PGLS, ACACA, and PDHA1 genes play vital roles in connecting anaerobic glycolytic signaling with its branch pathways (Fig. S12), while the up-regulation of such genes revealed that GExos may also activate the signaling network related to anaerobic glycolysis to activate the migration and vessel formation ability of HUVECs (Fig. S13 and Fig. 4C).

The transcriptome results suggest the possible regulatory effect of GExos on glucose metabolism under an HG environment (Fig. 4D). Consequently, we further investigated the specific effects of GExos on glucose metabolites in HUVECs (Fig. S14). Consequently, 271 and 103 metabolites were shown to be up- and down-regulated in GExos-treated HUVECs, respectively (Fig. 4E and Fig. S15). As expected, the differentially expressed metabolites significantly enriched in the signaling relevant to glycolysis, citrate cycle, etc. (Fig. 4F and G). The joint analysis of all differentially expressed metabolites and 6 glycolysis-related genes (PFKM, PGK1, ENO1, etc.) is shown in Fig. 4H. It was confirmed that the levels of metabolic intermediates in anaerobic glycolysis and key molecules in oxidative phosphorylation were significantly regulated by GExos (Fig. 4I_1_ to I_3_). In the HG+GExos group, glucose 6-phosphate (G6P) was down-regulated to 70%, fructose 1,6-bisphosphate (FDP) was significantly up-regulated by 2.10-fold, and the rate of G6P conversion to FDP represents the flux of anaerobic glycolysis. While the content of acetyl-CoA in HG+GExos was significantly down-regulated to 60% of the HG group (Fig. 4J_1_ to J_3_), the level of acetyl-CoA represents the flux of oxidative phosphorylation.

The changes in representative genes and metabolites reveal that GExos hold great potential to alleviate HG-induced endothelial dysfunction and promote angiogenesis by reprogramming glycolysis of HUVECs. In detail, on the one hand, GExos up-regulate the crucial early step of glycolysis, the anaerobic glycolysis process, and the up-regulated PFKM gene by GExos encodes for enzyme phosphofructokinase, which significantly promotes the conversion rate of G6P to FDP. The incorporation of FDP can act as an energy substrate for the improvement of ATP availability (Fig. S16) and decrease oxidative stress by limiting free radical production and improving antioxidant systems [49]. On the other hand, GExos are likely to attenuate the later step of glycolysis, the oxidative phosphorylation process, by down-regulating acetyl-CoA, which directly reduces ROS production of HUVECs under HG conditions (Fig. 3G).

### In vivo angiogenic efficacy and mechanism of GExos in a diabetic ulcer model

In vivo, hyperglycemia in diabetes particularly affects the endothelial functions that induce non-angiogenesis, perpetuating the development and persistence of chronic ulcers. Therefore, to evaluate the in vivo angiogenic potential of GExos in HG-conditioned diseases, the diabetic animal model, B6.BKS(D)-*Lepr ^db^*/J (*db/db*) mouse strain, which exhibits the features of typical spontaneous hyperglycemia (Fig. S17) [50], was utilized. In particular, 8- to 12-week-old male *db* mice were randomly selected, and a full-thickness skin wound 12 mm in diameter was created and fixed with a circular ring to represent the diabetic skin ulcer (Fig. S18). Considering that diabetic ulcers are severe local complications of diabetes mellitus, GExos suspension was applied in situ to the wound site, making direct contact with the diseased cells for the local treatment of diabetic ulcers. This in vivo route of administration can also circumvent the issues related to first-pass effects and rapid drug degradation.

**Fig. 5.**
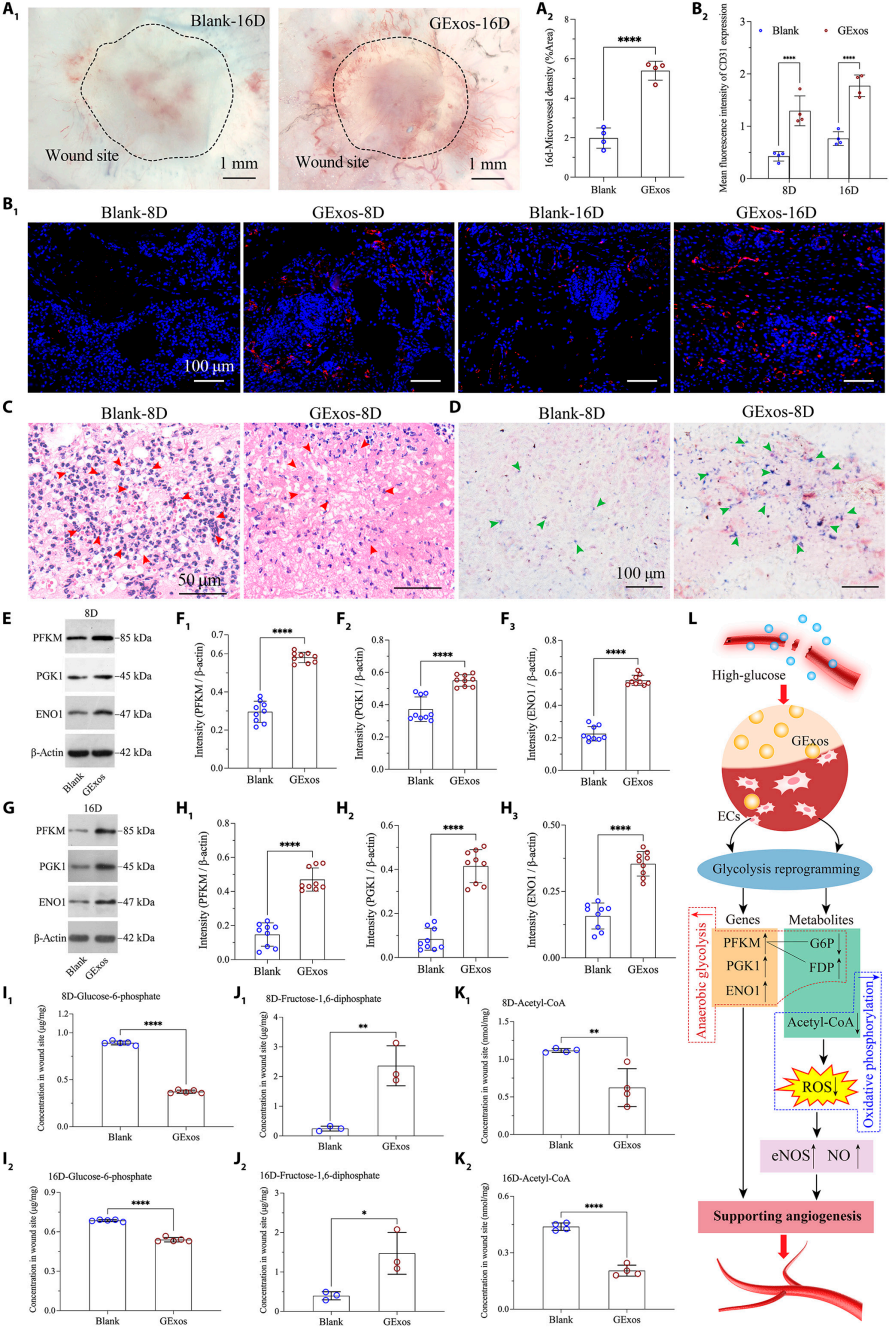
GExos promote angiogenesis in diabetic ulcers in mice by reprogramming glycolysis. Microvascular imaging (A_1_) and semi-quantification of the microvascular density (A_2_) in diabetic healed skins. 8D, 8 days; 16D, 16 days. Immunofluorescence staining (B_1_) of CD31 (red) and semi-quantification of the area percentage of CD31 (B_2_) in diabetic healed skins. H&E staining (C) and eNOS staining (D) in diabetic healed skins. Expression levels (E) and quantitative analysis (F_1_ to F_3_) of PFKM, PGK1, and ENO1 on day 8 in diabetic healed skin. Expression levels (G) and quantitative analysis (H_1_ to H_3_) of PFKM, PGK1, and ENO1 on day 16 in diabetic healed skin. Quantitative analysis of G6P (I_1_ and I_2_), FDP (J_1_ and J_2_), and Acetyl-CoA (K_1_ and K_2_) in diabetic healed skins on day 8 and day 16. (L) Schematic diagram of the therapeutic mechanism of GExos promoting angiogenesis in diabetic ulcers. *P* values are shown: *P < 0.05, **P < 0.01, ****P < 0.0001.

We then evaluated the pro-angiogenic ability of GExos in diabetic wound sites. In particular, the microimaging of the dermis shows that the development and formation of microvascular networks were clearly found in the wounded skins of the GExos group on day 16 (Fig. 5A_1_), and the microvascular formation density was 2.72-fold higher than that of the blank group (Fig. 5A_2_). The CD31 immunofluorescence staining was utilized to quantitatively evaluate angiogenesis at the ulcer wound modeling site due to the clarity with which fluorescent signals illustrate the location and distribution of target proteins. The images of CD31 monochromatic fluorescence channels are presented in Fig. S19. It can be seen that GExos treatment resulted in a substantial increase in the CD31 expression on day 8 and day 16 (Fig. 5B_1_), which was 1.66- and 2.32-fold higher than those of the blank groups, respectively (Fig. 5B_2_). These results demonstrated the amazing curative effect of GExos in promoting microvascular angiogenesis and nascent vessel network reconstruction in diabetic hyperglycemia diseases.

The level of inflammatory cell infiltration serves as an important reflection of the in vivo oxidative stress in diabetic ulcers. The excessive or prolonged inflammatory state induced by persistent hyperglycemia often leads to the suppression of angiogenic responses, contributing to impaired wound healing in diabetes, which is the reason for the significant inflammatory cell infiltration, and less matrix formation was observed in the wounded skin of mice in the blank group at the early stage (Fig. 5C). By contrast, the inflammatory cell density in the GExos-treated group was only 57% of that of the blank group, and the healing matrix was well regenerated (Fig. S20), suggesting that GExos reduce oxidative stress in diabetic ulcers. In diabetic conditions, increased oxidative stress can lead to reduced eNOS expression and activity. Remarkably, compared to the blank group mice, a large amount of eNOS expression was also detected in the early healing point of GExos-treated diabetic wounds (Fig. 5D). In summary, these findings further underline the important roles of GExos in the regulation of reducing oxidative stress and enhancing eNOS level to promote angiogenesis in diabetic ulcers.

Mechanistically, based on the in vitro results (Fig. 4), the evolution of vital gene expression and metabolites in the nascent skins during diabetic ulcer healing was further verified. It was found that, compared to the blank group, the expression levels of PFKM, PGK1, and ENO1 in the GExos-treated group were significantly increased by 1.96-, 1.48-, and 2.46-fold on day 8 (Fig. 5E and F_1_ to F_3_), and by 3.19-, 4.98-, and 2.25-fold on day 16 (Fig. 5G and H_1_ to H_3_), respectively. PFKM, PGK1, and ENO1 have been reported for their efficiency in up-regulating the anaerobic glycolysis process to provide biomass and energy for angiogenesis [46–48]. Besides, in the regulation of glycolysis metabolites, the content of G6P in the GExos-treated group was significantly down-regulated to 42% and 79% on day 8 and day 16, respectively (Fig. 5I_1_ and I_2_). The content of FDP in the GExos-treated group was significantly up-regulated on days 8 and 16 by 9.62- and 3.72-fold, respectively (Fig. 5J_1_ and J_2_). The content of acetyl-CoA in the GExos-treated group was significantly down-regulated to 56% and 47% on days 8 and 16, respectively (Fig. 5K_1_ and K_2_). This is consistent with the results of in vitro experiments, which jointly indicate that GExos significantly promote the conversion rate of G6P to FDP to enhance anaerobic glycolysis, while inhibiting the generation of acetyl-CoA to attenuate the oxidative phosphorylation, thereby reducing the oxidative stress to restore the angiogenesis ability of dysfunctional ECs in diabetic ulcers (Fig. 5L).

### GExos accelerate the healing of diabetic skin ulcers with high biosafety

**Fig. 6.**
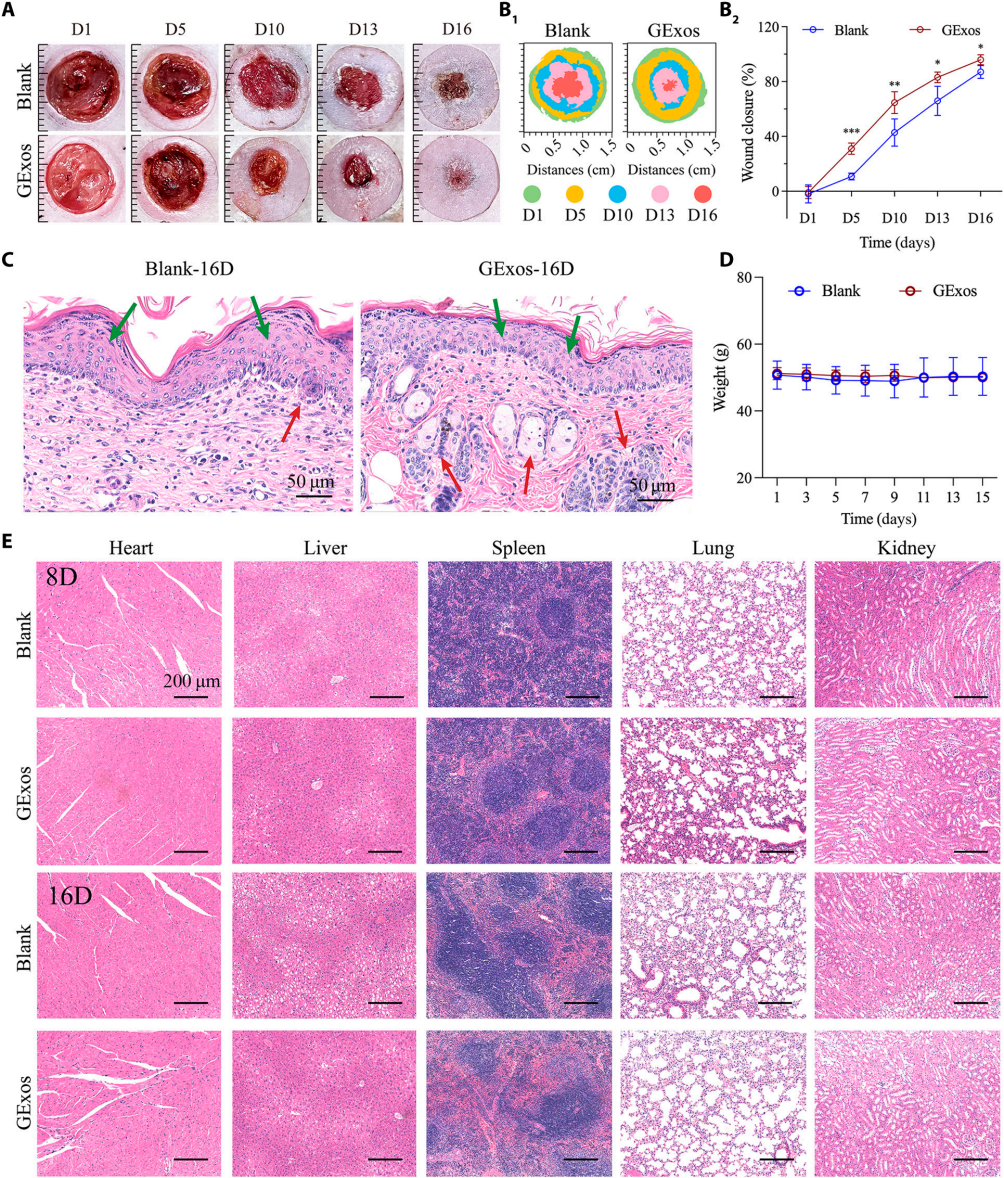
GExos promote diabetic chronic ulcer healing with high biosafety. (A) Photographs of the wound appearance from day 1 to day 16 post-wounding of the Blank and GExos groups. Visualization (B_1_) and quantitative analysis (B_2_) of wound healing ratio over time. (C) H&E staining of the diabetic healed skins on day 16 in Blank and GExos groups. The green arrows indicate the epidermis and the red arrows indicate the skin appendages. (D) Body weight of rats at different time points. (E) Images of H&E-stained organ slices of the tested animals on day 8 and day 16, respectively. *P* values are shown: *P < 0.05, **P < 0.01, ***P < 0.001.

Angiogenesis followed by blood vessel formation is one of the most important activities that determine the healing of diabetic ulcers [51,52]. Based on the unique angiogenic effects of GExos in both normal and hyperglycemia conditions, the influence of GExos in diabetic wound healing was further evaluated. As Fig. 6A and B show, all of the wounds expressed gradual healing. The GExos-treated *db* mice group showed a significantly faster healing rate throughout the experiment period than the blank group, while the wounds of the 200 μg/ml GExos group showed the highest healing rates throughout the testing period (Fig. 6A and Figs. S21 and S22). Consequently, 200 μg/ml was thus selected as the optimal dosage for all the in vivo experiments.

By H&E staining, the histological difference of the tested samples was indicated, as thinner epidermis with more regenerated skin appendages was identified in the GExos group. By contrast, thickened epidermis indicating scarlike characteristics and missing skin appendages were observed in the blank group (Fig. 6C). These results give further evidence of the regenerative effects of GExos treatment in diabetic skin ulcers, partly contributing to its vigorous angiogenic effects in the ECs in a hyperglycemia environment. As regards biosafety, the body weights of all tested mice remained stable throughout the experimental period (Fig. 6D), and there was no pathological change in the major organs of the tested mice (Fig. 6E), demonstrating the good biocompatibility of GExos for both in vitro and in vivo applications.

## Discussion

The clinical utilization of angiogenic drugs in an HG environment, encompassing angiopoietins, growth factors, and protein kinase C inhibitors, typically entails side effects such as local allergic reactions and immunological risks. The synthesized nanodrugs have recently gained attention in promoting angiogenesis; however, most of them encounter persistent challenges related to degradation difficulties, compromised biocompatibility, and limited efficiency in loading or releasing external molecules. Moreover, few studies have reported the angiogenic potential of these nanotherapeutics under HG conditions and diabetic hyperglycemia.

For the first time, this study demonstrates the efficacy of GExos in transferring functional substances to ECs initiating vigorous angiogenic effects under HG conditions. The interaction network between the mRNAs of ECs and the delivered miRNAs of GExos shows that GExos efficiently transferred the incorporated nucleic acids with therapeutic effects to enhance the glycolysis reprogramming-mediated angiogenesis under HG conditions (Fig. S23). In contrast to most reported chemical synthetic nanomaterials, the medicinal plant-derived exosomes have the advantages of abundant resources, simple fabrication, fast and complete degradation, and less immunological risk, and their incorporated active substances often exhibit surprising therapeutic effects [53]. All these distinctive characteristics highlight that medicinal plant-derived exosomes are capable of overcoming cellular membrane barriers, enabling the transfer of the incorporated biosubstance into target cells for disease therapy [54,55].

Furthermore, our study reveals that one of the main molecular mechanisms for the angiogenic effects of GExos in ECs can be attributed to the glycolysis reprogramming regulation by GExos. We found that GExos up-regulate a crucial early step of glycolysis, the anaerobic glycolysis process that promotes the proliferation, migration, and tubule formation of ECs. Meanwhile, GExos attenuate a later step of glycolysis, the oxidative phosphorylation process to reverse endothelial dysfunction under HG conditions. To date, almost all available pro-angiogenic therapies have been focused on the growth factor pathways. By contrast, GExos directly target the glucose metabolism of ECs in an HG environment, representing a novel and attractive therapeutic method of vascular disease therapy in diabetic hyperglycemia with high biosafety.

In conclusion, GExos have exhibited remarkable efficacy as novel nanotherapeutics in transferring the encapsulated bioactive substances to ECs, which markedly ameliorate the endothelial dysfunction and stimulate glycolysis reprogramming-mediated angiogenesis by up-regulating the anaerobic glycolysis and down-regulating the oxidative phosphorylation under HG conditions. GExos represent a novel paradigm in engineering medicinal plant-derived exosomes into the next generation of nanotherapeutics for vascular disease therapy associated with diabetic hyperglycemia.

## Data Availability

The datasets used and/or analyzed during the current study are available from the corresponding author upon reasonable request.

## References

[1] De Vriese AS, Verbeuren TJ, Van De Voorde J, Lameire NH, Vanhoutte PM. Endothelial dysfunction in diabetes. Br J Pharmacol. 2000;130(5):963–974. 10882379 10.1038/sj.bjp.0703393 PMC1572156

[2] Hink U, Li H, Mollnau H, Oelze M, Matheis E, Hartmann M, Skatchkov M, Thaiss F, Stahl RAK, Warnholtz A, et al. Mechanisms underlying endothelial dysfunction in diabetes mellitus. Circ Res. 2001;88(2):E14–E22. 11157681 10.1161/01.res.88.2.e14

[3] Yan LJ. Pathogenesis of chronic hyperglycemia: From reductive stress to oxidative stress. J Diabetes Res. 2014;2014: Article 137919. 25019091 10.1155/2014/137919 PMC4082845

[4] Kolluru GK, Bir SC, Kevil CG. Endothelial dysfunction and diabetes: Effects on angiogenesis, vascular remodeling, and wound healing. Int J Vasc Med. 2012;2012: Article 918267. 22611498 10.1155/2012/918267 PMC3348526

[5] Li T, Zhang T. The application of nanomaterials in angiogenesis. Curr Stem Cell Res Ther. 2020;16(1):74–82. 10.2174/1574888X15666200211102203 32066364

[6] Yoshida YG, Yan S, Xu H, Yang J. Novel metal nanomaterials to promote angiogenesis in tissue regeneration. Eng Regen. 2023;4(3):265–276. 37234753 10.1016/j.engreg.2023.03.008 PMC10207714

[7] Kargozar S, Baino F, Hamzehlou S, Hamblin MR, Mozafari M. Nanotechnology for angiogenesis: Opportunities and challenges. Chem Soc Rev. 2020;49(14):5008–5057. 32538379 10.1039/c8cs01021h PMC7418030

[8] De Vriese AS, Verbeuren TJ, Van De Voorde J, Lameire NH, Vanhoutte PM. Endothelial dysfunction in diabetes. Br J Pharmacol. 2000;130(5):963–974. 10882379 10.1038/sj.bjp.0703393 PMC1572156

[9] Cui L, Liang J, Liu H, Zhang K, Li J. Nanomaterials for angiogenesis in skin tissue engineering. Tissue Eng Part B Rev. 2020;26(3):203–216. 31964266 10.1089/ten.TEB.2019.0337

[10] Wietecha MS, DiPietro LA. Therapeutic approaches to the regulation of wound angiogenesis. Adv Wound Care. 2013;2(3):81–86. 10.1089/wound.2011.0348 PMC3623575 24527330

[11] Sharifi S, Hajipour MJ, Gould L, Mahmoudi M. Nanomedicine in healing chronic wounds: Opportunities and challenges. Mol Pharm. 2021;18(2):550–575. 32519875 10.1021/acs.molpharmaceut.0c00346

[12] Alagarsamy KN, Mathan S, Yan W, Rafieerad A, Sekaran S, Manego H, Dhingra S. Carbon nanomaterials for cardiovascular theranostics: Promises and challenges. Bioact Mater. 2021;6(8):2261–2280. 33553814 10.1016/j.bioactmat.2020.12.030 PMC7829079

[13] Herrmann IK, Wood MJA, Fuhrmann G. Extracellular vesicles as a next-generation drug delivery platform. Nat Nanotechnol. 2021;16(7):748–759. 34211166 10.1038/s41565-021-00931-2

[14] Van Niel G, D’Angelo G, Raposo G. Shedding light on the cell biology of extracellular vesicles. Nat Rev Mol Cell Biol. 2018;19(4):213–228. 29339798 10.1038/nrm.2017.125

[15] El Andaloussi S, Mäger I, Breakefield XO, Wood MJA. Extracellular vesicles: Biology and emerging therapeutic opportunities. Nat Rev Drug Discov. 2013;12(5):347–357. 23584393 10.1038/nrd3978

[16] Di Raimo R, Mizzoni D, Spada M, Dolo V, Fais S, Logozzi M. Oral treatment with plant-derived exosomes restores redox balance in H_2_O_2_-treated mice. Antioxidants. 2023;12(6):1169. 37371899 10.3390/antiox12061169 PMC10295262

[17] Castelli G, Logozzi M, Mizzoni D, Di Raimo R, Cerio A, Dolo V, Pasquini L, Screnci M, Ottone T, Testa U, et al. Ex vivo anti-leukemic effect of exosome-like grapefruit-derived nanovesicles from organic farming—The potential role of ascorbic acid. Int J Mol Sci. 2023;24(21):15663. 37958646 10.3390/ijms242115663 PMC10648274

[18] Yan G, Xiao Q, Zhao J, Chen H, Xu Y, Tan M, Peng L. *Brucea javanica* derived exosome-like nanovesicles deliver miRNAs for cancer therapy. J Control Release. 2024;367:425–440. 38295998 10.1016/j.jconrel.2024.01.060

[19] Tan M, Xu W, Yan G, Xu Y, Xiao Q, Liu A, Peng L. Oriented artificial niche provides physical-biochemical stimulations for rapid nerve regeneration. Mater Today Bio. 2023;22: Article 100736. 10.1016/j.mtbio.2023.100736 PMC10374615 37521524

[20] Logozzi M, Di Raimo R, Mizzoni D, Fais S. The potentiality of plant-derived nanovesicles in human health—A comparison with human exosomes and artificial nanoparticles. Int J Mol Sci. 2022;23(9):4919. 35563310 10.3390/ijms23094919 PMC9101147

[21] Dad HA, Gu TW, Zhu AQ, Huang LQ, Peng LH. Plant exosome-like nanovesicles: Emerging therapeutics and drug delivery nanoplatforms. Mol Ther. 2021;29(1):13–31. 33278566 10.1016/j.ymthe.2020.11.030 PMC7791080

[22] Kalluri R, LeBleu VS. The biology, function, and biomedical applications of exosomes. Science. 2020;367(6478): Article eaau6977. 32029601 10.1126/science.aau6977 PMC7717626

[23] Sengupta S, Toh SA, Sellers LA, Skepper JN, Koolwijk P, Leung HW, Yeung HW, Wong RNS, Sasisekharan R, Fan TPD. Modulating angiogenesis: The yin and the yang in ginseng. Circulation. 2004;110(10):1219–1225. 15337705 10.1161/01.CIR.0000140676.88412.CF

[24] Morisaki N, Watanabe S, Tezuka M, Zenibayashi M, Shüna R, Koyama N, Kanzaki T, Saito Y. Mechanism of angiogenic effects of saponin from ginseng *Radix rubra* in human umbilical vein endothelial cells. Br J Pharmacol. 1995;115(7):1188–1193. 7582543 10.1111/j.1476-5381.1995.tb15023.x PMC1908790

[25] Huang L, Cai HA, Zhang MS, Liao RY, Huang X, Hu FD. Ginsenoside Rg1 promoted the wound healing in diabetic foot ulcers via miR-489–3p/Sirt1 axis. J Pharmacol Sci. 2021;147(3):271–283. 34507636 10.1016/j.jphs.2021.07.008

[26] Zhang J, Liu M, Huang M, Chen M, Zhang D, Luo L, Ye G, Deng L, Peng Y, Wu X, et al. Ginsenoside F1 promotes angiogenesis by activating the IGF-1/IGF1R pathway. Pharmacol Res. 2019;144:292–305. 31048033 10.1016/j.phrs.2019.04.021

[27] Tang K, Qin W, Wei R, Jiang Y, Fan L, Wang Z, Tan N. Ginsenoside Rd ameliorates high glucose-induced retinal endothelial injury through AMPK-STRT1 interdependence. Pharmacol Res. 2022;179: Article 106123. 35150861 10.1016/j.phrs.2022.106123

[28] Xu XH, Yuan TJ, Dad HA, Shi MY, Huang YY, Jiang ZH, Peng LH. Plant exosomes as novel nanoplatforms for microRNA transfer stimulate neural differentiation of stem cells in vitro and in vivo. Nano Lett. 2021;21(19):8151–8159. 34586821 10.1021/acs.nanolett.1c02530

[29] Subramanian A, Tamayo P, Mootha VK, Mukherjee S, Ebert BL, Gillette MA, Paulovich A, Pomeroy SL, Golub TR, Lander ES, et al. Gene set enrichment analysis: A knowledge-based approach for interpreting genome-wide expression profiles. Proc Natl Acad Sci U S A. 2005;102(43):15545–15550. 16199517 10.1073/pnas.0506580102 PMC1239896

[30] Mootha VK, Lindgren CM, Eriksson KF, Subramanian A, Sihag S, Lehar J, Puigserver P, Carlsson E, Ridderstråle M, Laurila E, et al. PGC-1α-responsive genes involved in oxidative phosphorylation are coordinately downregulated in human diabetes. Nat Genet. 2003;34(3):267–273. 12808457 10.1038/ng1180

[31] Van den Boorn JG, Daßler J, Coch C, Schlee M, Hartmann G. Exosomes as nucleic acid nanocarriers. Adv Drug Deliv Rev. 2013;65(3):331–335. 22750807 10.1016/j.addr.2012.06.011

[32] Zhao Q, Wang T, Wang H, Cao P, Jiang C, Qiao H, Peng L, Lin X, Jiang Y, Jin H. et al., Consensus statement on research and application of Chinese herbal medicine derived extracellular vesicles-like particles (2023 edition). Chinese Herb Med. 2024;16(1):3–12. 10.1016/j.chmed.2023.11.002 PMC10874762 38375050

[33] Chen X, Lin Y, Hu Y, Liu C, Lan K, Jia W. Phytochemistry, metabolism, and metabolomics of ginseng. Chinese Herb Med. 2015;7(2):98–108.

[34] Mendes ND, Freitas AT, Sagot MF. Survey and summary: Current tools for the identification of miRNA genes and their targets. Nucleic Acids Res. 2009;37(8):2419–2433. 19295136 10.1093/nar/gkp145 PMC2677885

[35] Tenchov R, Sasso JM, Wang X, Liaw W-S, Chen C-A, Zhou QA. Exosomes—Nature’s lipid nanoparticles, a rising star in drug delivery and diagnostics. ACS Nano. 2022;16(11):17802–17846. 36354238 10.1021/acsnano.2c08774 PMC9706680

[36] Beckman JA, Creager MA, Libby P. Diabetes and atherosclerosis epidemiology, pathophysiology, and management. JAMA. 2002;287(19):2570–2581. 12020339 10.1001/jama.287.19.2570

[37] Zhan Q, Yi K, Li X, Cui X, Yang E, Chen N, Yuan X, Zhao J, Hou X, Kang C. Phosphatidylcholine-engineered exosomes for enhanced tumor cell uptake and intracellular antitumor drug delivery. Macromol Biosci. 2021;21(8): Article e2100042. 33949800 10.1002/mabi.202100042

[38] Hannun YA, Obeid LM. Sphingolipids and their metabolism in physiology and disease. Nat Rev Mol Cell Biol. 2018;19(3):175–191. 29165427 10.1038/nrm.2017.107 PMC5902181

[39] Ke W, Afonin KA. Exosomes as natural delivery carriers for programmable therapeutic nucleic acid nanoparticles (NANPs). Adv Drug Deliv Rev. 2021;176: Article 113835. 34144087 10.1016/j.addr.2021.113835 PMC8440450

[40] Caporali A, Emanueli C. MicroRNA regulation in angiogenesis. Vasc Pharmacol. 2011;55(4):79–86. 10.1016/j.vph.2011.06.006 21777698

[41] Yao J, Wu XY, Yu Q, Yang SF, Yuan J, Zhang ZQ, Xue JS, Jiang Q, Chen MB, Xue GH, et al. The requirement of phosphoenolpyruvate carboxykinase 1 for angiogenesis in vitro and in vivo. Sci Adv. 2022;8(21):6928. 10.1126/sciadv.abn6928 PMC9140980 35622925

[42] Huang J, Yang R, Jiao J, Li Z, Wang P, Liu Y, Li S, Chen C, Li Z, Qu G, et al. A click chemistry-mediated all-peptide cell printing hydrogel platform for diabetic wound healing. Nat Commun. 2023;14(1):7856. 38030636 10.1038/s41467-023-43364-2 PMC10687272

[43] Guo Y, Ding S, Shang C, Zhang C, Li M, Zhang Q, Gu L, Heng BC, Zhang S, Mei F, et al. Multifunctional PtCuTe nanosheets with strong ROS scavenging and ROS-independent antibacterial properties promote diabetic wound healing. Adv Mater. 2023;36(8): Article e2306292. 37723937 10.1002/adma.202306292

[44] Falkenberg KD, Rohlenova K, Luo Y, Carmeliet P. The metabolic engine of endothelial cells. Nat Metab. 2019;1(10):937–946. 32694836 10.1038/s42255-019-0117-9

[45] De Bock K, Georgiadou M, Carmeliet P. Role of endothelial cell metabolism in vessel sprouting. Cell Metab. 2013;18(5):634–647. 23973331 10.1016/j.cmet.2013.08.001

[46] Giallongo A, Oliva D, Calì L, Barba G, Barbieri G, Feo S. Structure of the human gene for α-enolase. Eur J Biochem. 1990;190(3):567–573. 2373081 10.1111/j.1432-1033.1990.tb15611.x

[47] Pan CH, Chien YC, Sung MS, Huang HY, Sheu MJ, Wu CH. Pathological role of phosphoglycerate kinase 1 in balloon angioplasty-induced neointima formation. Int J Mol Sci. 2021;22(16):8822. 34445528 10.3390/ijms22168822 PMC8396187

[48] Zhou Y, Lin F, Wan T, Chen A, Wang H, Jiang B, Zhao W, Liao S, Wang S, Li G, et al. ZEB1 enhances Warburg effect to facilitate tumorigenesis and metastasis of HCC by transcriptionally activating PFKM. Theranostics. 2021;11(12):5926–5938. 33897890 10.7150/thno.56490 PMC8058737

[49] Alva N, Alva R, Carbonell T. Fructose 1,6-bisphosphate: A summary of its cytoprotective mechanism. Curr Med Chem. 2016;23(39):4396–4417. 27758716 10.2174/0929867323666161014144250

[50] Burke SJ, Batdorf HM, Burk DH, Noland RC, Eder AE, Boulos MS, Karlstad MD, Jason Collier J. Db/db mice exhibit features of human type 2 diabetes that are not present in weight-matched C57BL/6J mice fed a Western diet. J Diabetes Res. 2017;2017:8503754. 29038790 10.1155/2017/8503754 PMC5606106

[51] Bielefeld KA, Amini-Nik S, Alman BA. Cutaneous wound healing: Recruiting developmental pathways for regeneration. Cell Mol Life Sci. 2013;70(12):2059–2081. 23052205 10.1007/s00018-012-1152-9 PMC3663196

[52] Kumar P, Kumar S, Udupa EP, Kumar U, Rao P, Honnegowda T. Role of angiogenesis and angiogenic factors in acute and chronic wound healing. Plast Aesthetic Res. 2015;2:243–249.

[53] Yáñez-Mó M, Siljander PRM, Andreu Z, Zavec AB, Borràs FE, Buzas EI, Buzas K, Casal E, Cappello F, Carvalho J, et al. Biological properties of extracellular vesicles and their physiological functions. J Extracell Vesicles. 2015;4:27066. 25979354 10.3402/jev.v4.27066 PMC4433489

[54] Wang Q, Zhuang X, Mu J, Bin DZ, Jiang H, Xiang X, Xiang X, Wang B, Yan J, Miller D, et al. Delivery of therapeutic agents by nanoparticles made of grapefruit-derived lipids. Nat Commun. 2013;4:1867. 23695661 10.1038/ncomms2886 PMC4396627

[55] Ju S, Mu J, Dokland T, Zhuang X, Wang Q, Jiang H, Xiang X, Deng ZB, Wang B, Zhang L, et al. Grape exosome-like nanoparticles induce intestinal stem cells and protect mice from DSS-induced colitis. Mol Ther. 2013;21(7):1345–1357. 23752315 10.1038/mt.2013.64 PMC3702113

