# Supplementary material for: Plant-Derived Exosomes as Novel Nanotherapeutics Contrive Glycolysis Reprogramming-Mediated Angiogenesis for Diabetic Ulcer Healing
Source: Biomater Res. 2024 Jun 5;28:0035. doi: 10.34133/bmr.0035 (PMC11151174; doi:10.34133/bmr.0035)
Supplement: Supplementary 1 — Figs. S1 to S23 Tables S1 to S3 [file bmr.0035.f1.docx]

*Supporting Information*

**Plant-derived exosomes as novel nanotherapeutics contrive glycolysis reprogramming-mediated angiogenesis for diabetic ulcer healing**

Minhong Tan^1,3^, Yuda Liu^1^, Yang Xu^1^, Ge Yan^1^, Nan Zhou^1^, Haoran Chen^1^, Zhihong Jiang^2^, Lihua Peng^1,2^*

^1^College of Pharmaceutical Sciences, Zhejiang University, Hangzhou, 310058, PR China

^2^State Key Laboratory of Quality Research in Chinese Medicine, Macau University of Science and Technology, Macau, PR China

^3^School of Materials Science and Engineering, Zhejiang University, Hangzhou, 310027, PR China

*Address correspondence to

Lihua Peng, Ph.D., Associate Professor.

College of Pharmaceutical Sciences, Zhejiang University, 866# Yuhangtang Road, Hangzhou, 310058, P.R. China.

Email: [lhpeng@zju.edu.cn](mailto:lhpeng@zju.edu.cn)

Tel/Fax: +86-571-88981231

**This file includes:**

Supporting Figures S1 to S23

Supporting Tables S1 and S3


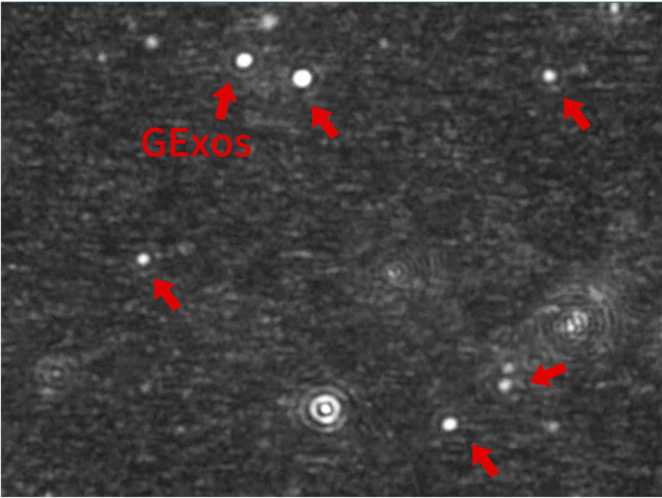


**Figure S1.** Nanoparticle tracking analysis and particles number quantification of GExos.


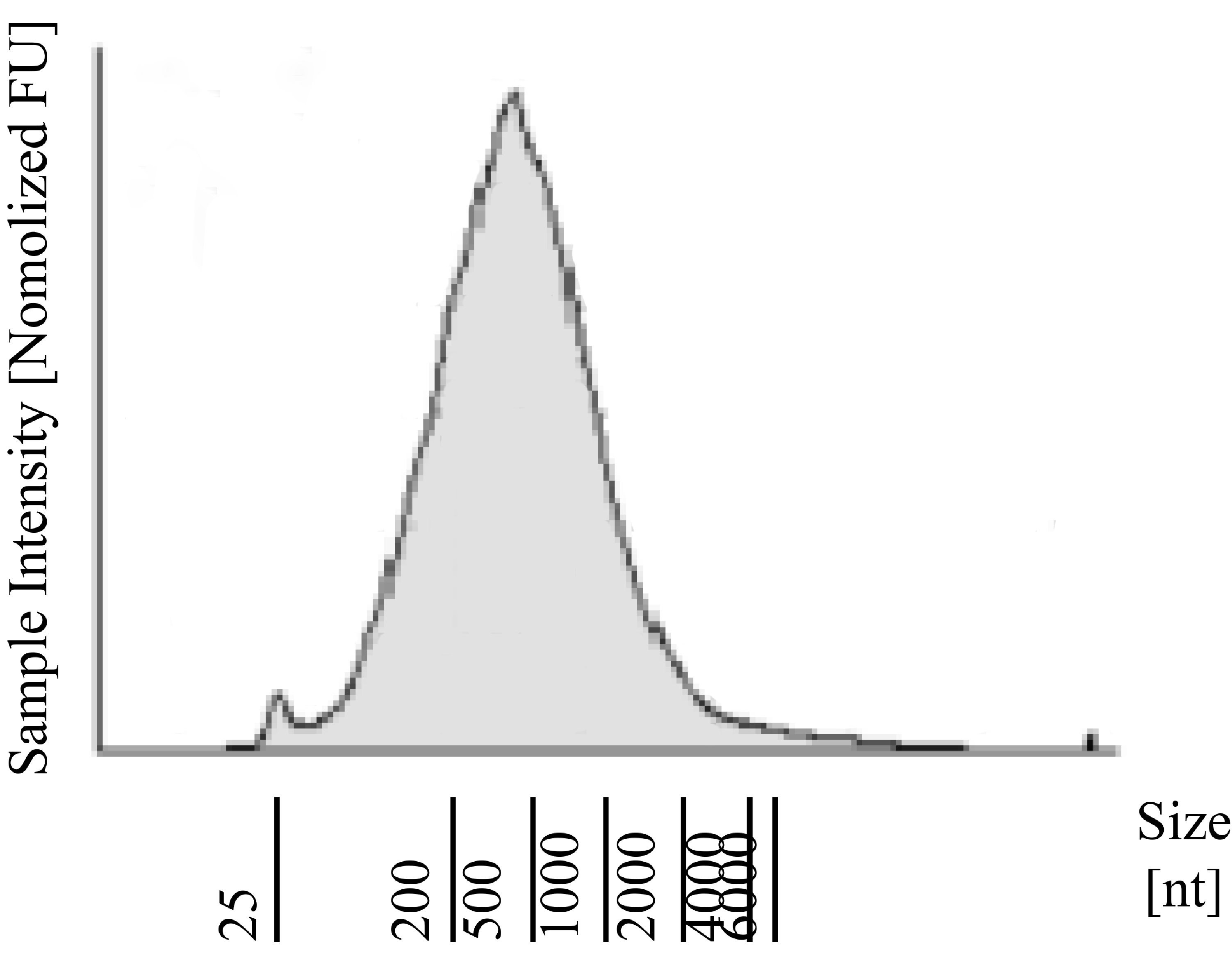


**Figure S2.** Length distribution of nucleic acids contained in GExos.


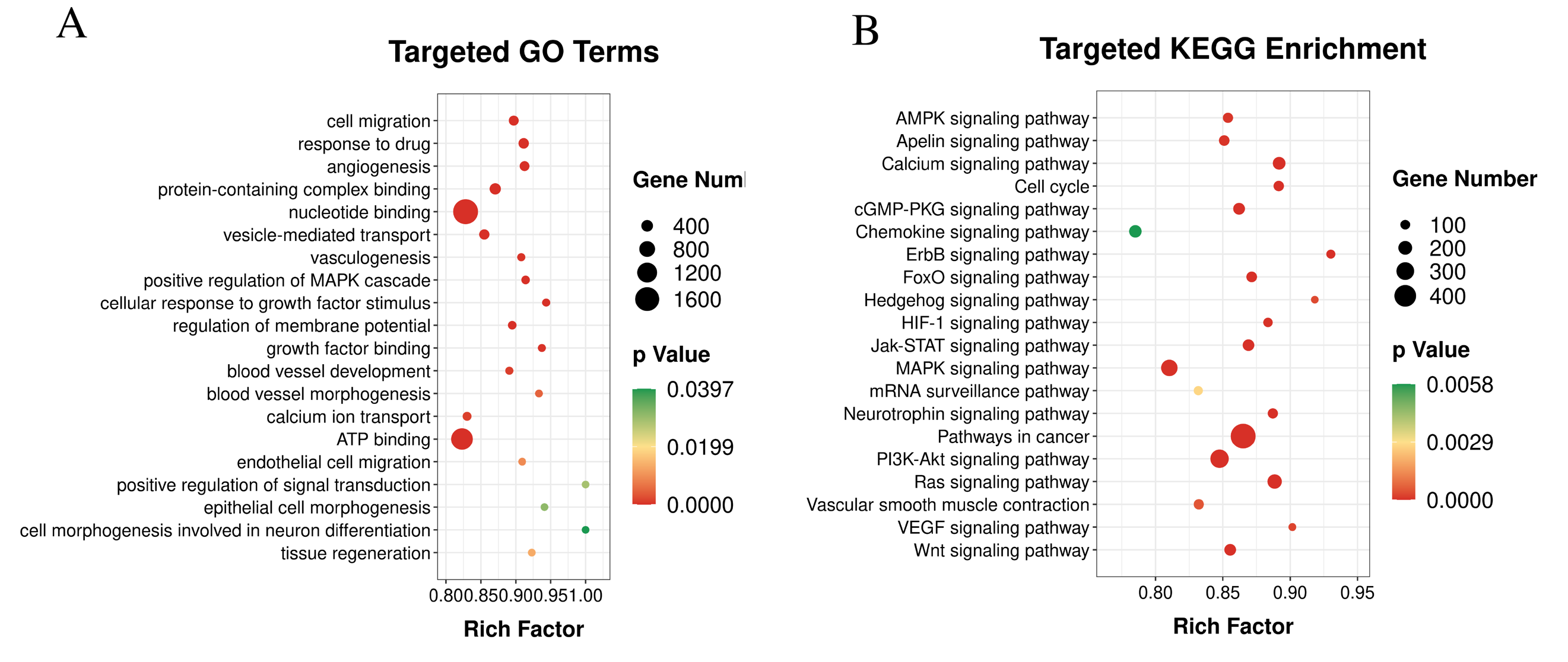


**Figure S3.** GO term and KEGG pathway enrichment analyses of the categories enriched in the specific target genes of GExos-miRNAs.


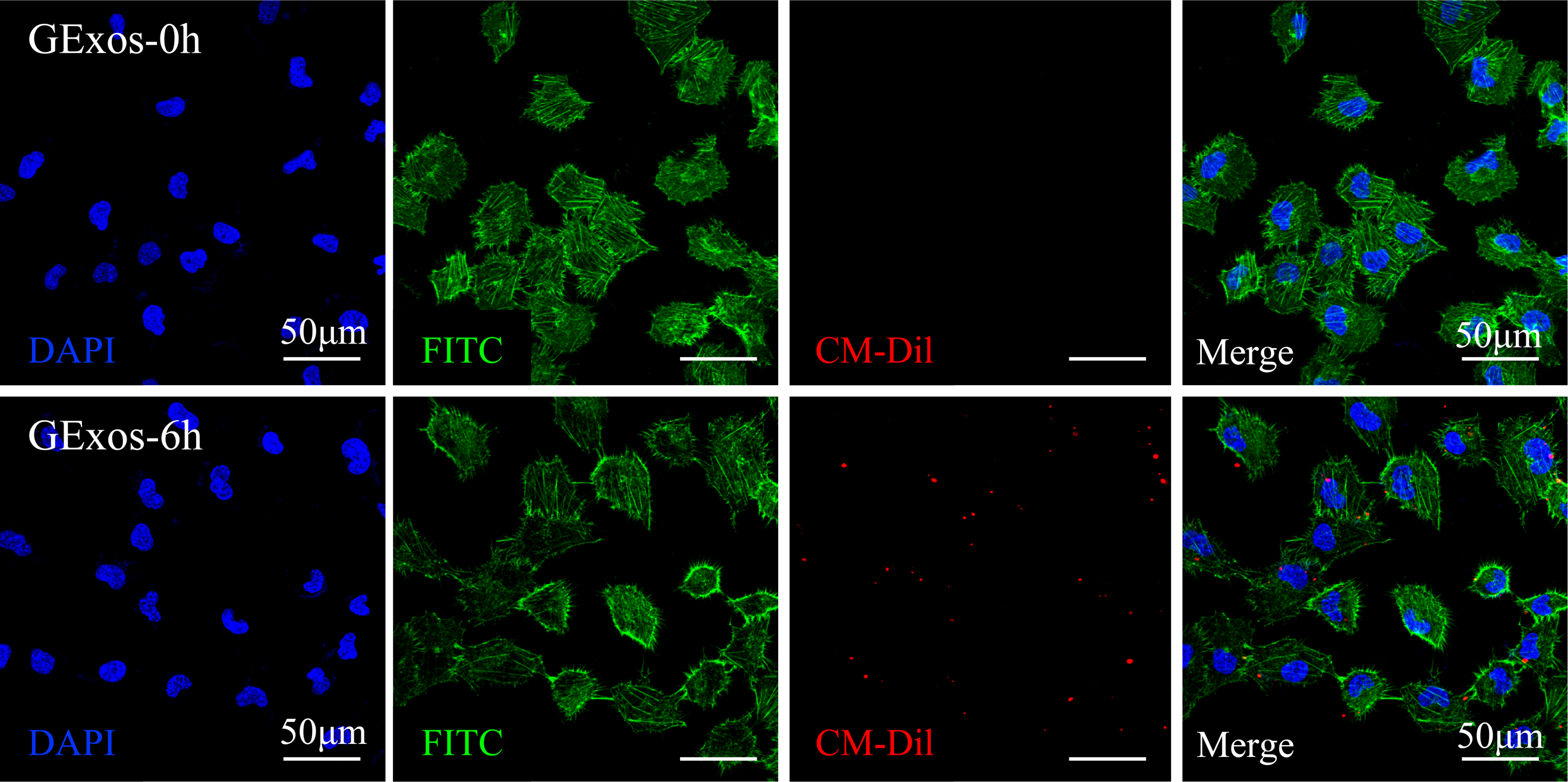


**Figure S4.** Uptake of CM-Dil (red) labeled GExos into HUVECs at 6 h after co-incubation. The nuclei and the cytoskeleton were stained with DAPI and FITC, respectively.


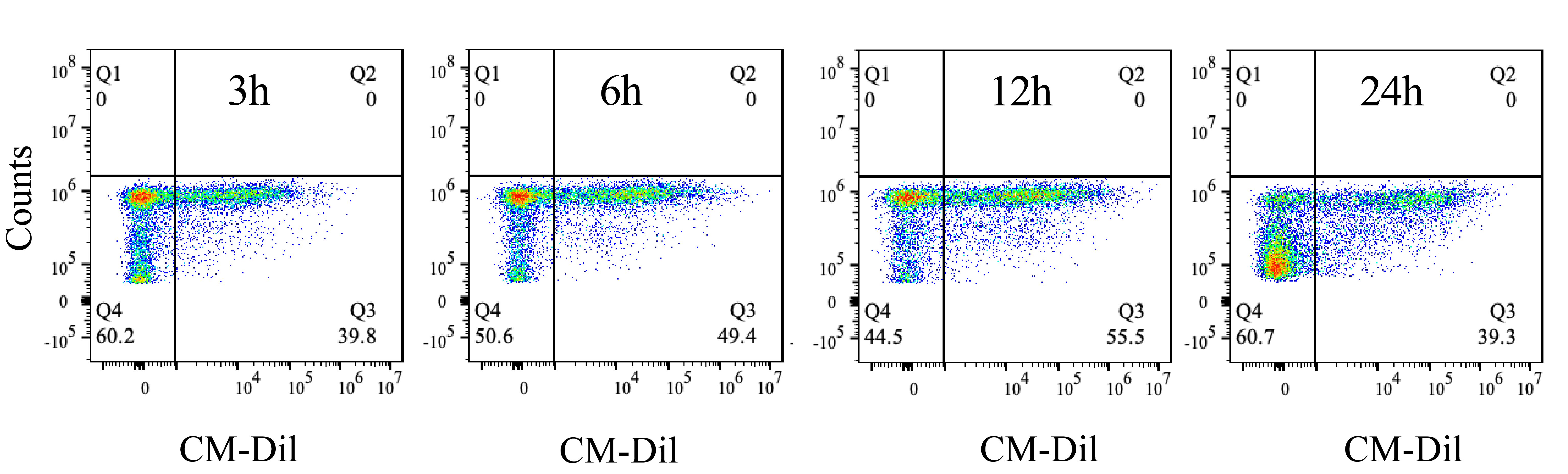


**Figure S5.** CM-Dil-positive rates of HUVECs co-cultured with the CM-Dil-labeled GExos for 0, 3, 6, 12, and 24 h, analyzed by flow cytometry.


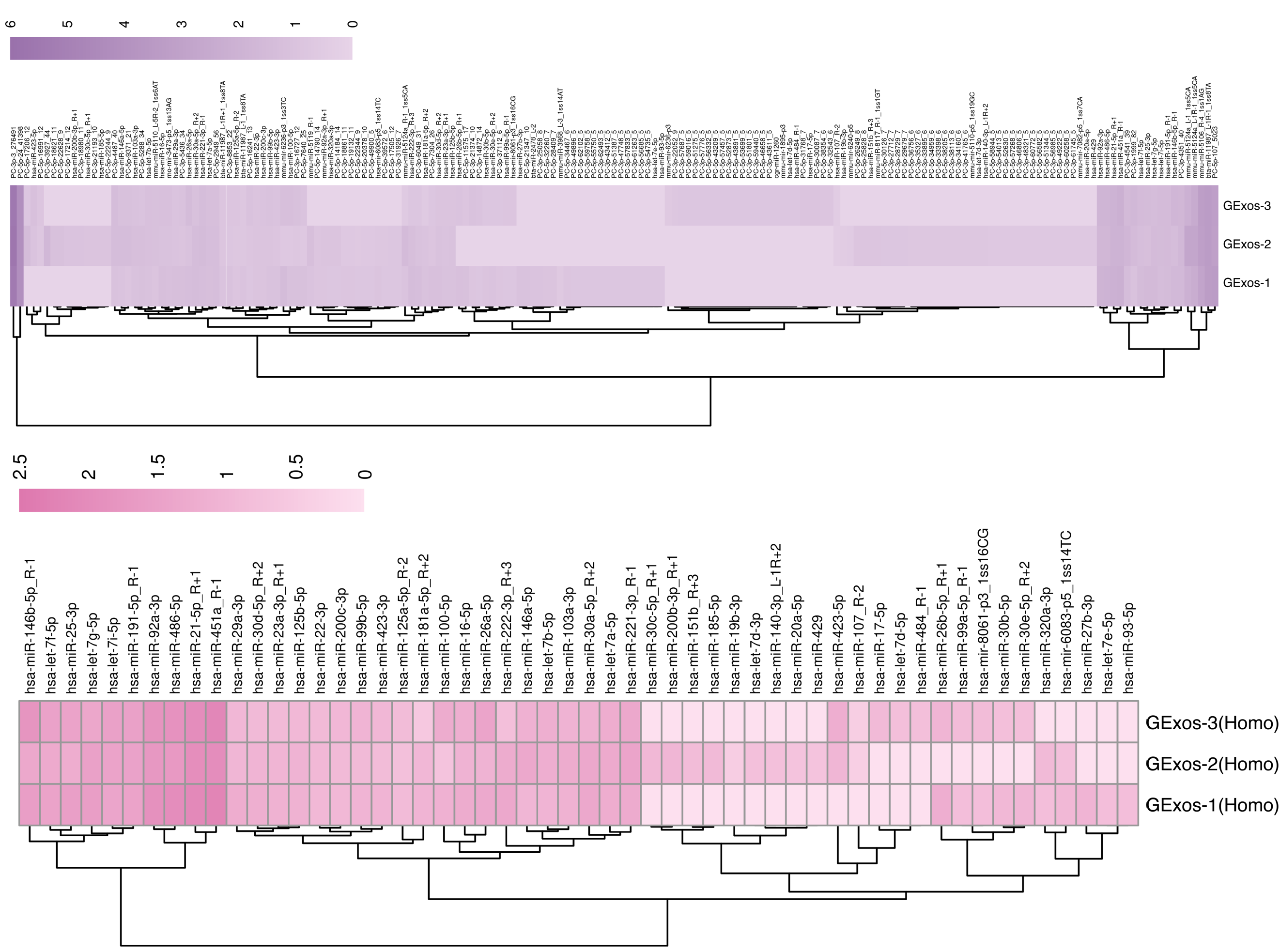


**Figure S6.** Heatmap of the expressed miRNA matched to the Homo genome database (and with gp1a class).


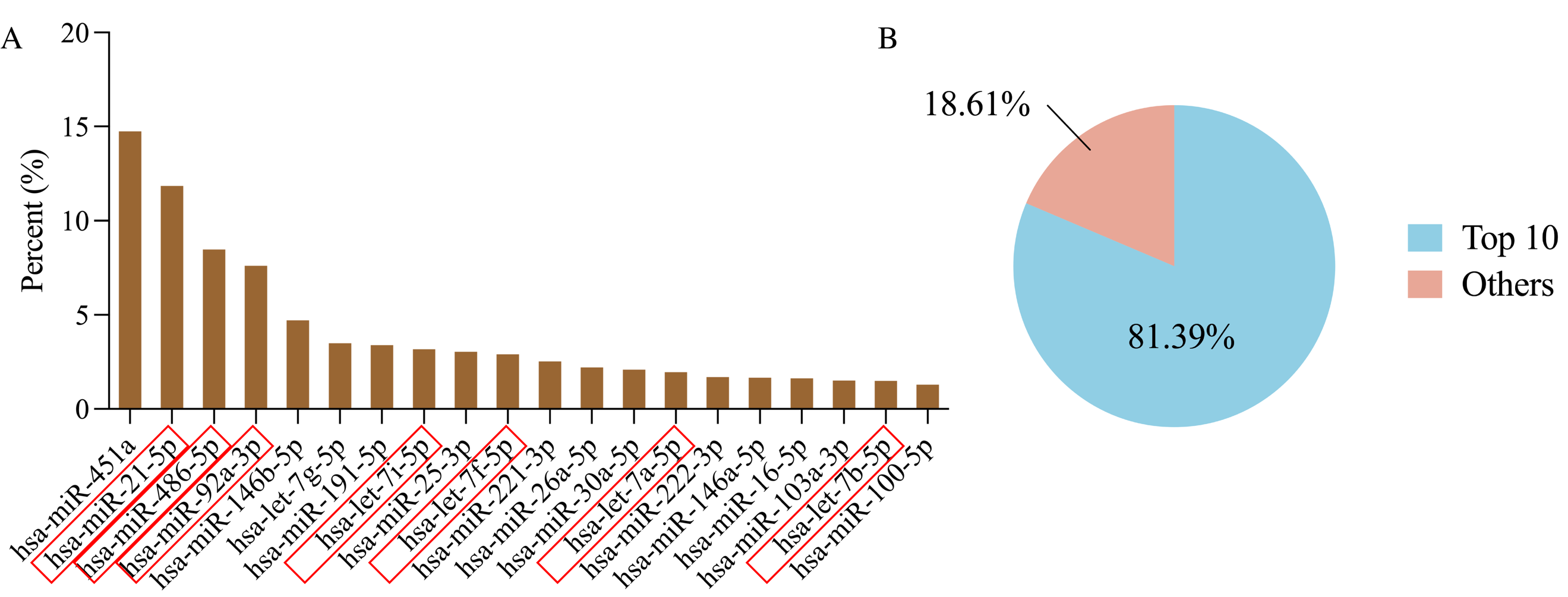


**Figure S7.** Normalized expression values (A) and total proportion of top 10 miRNAs (B) matched to the Homo genome database in GExos.


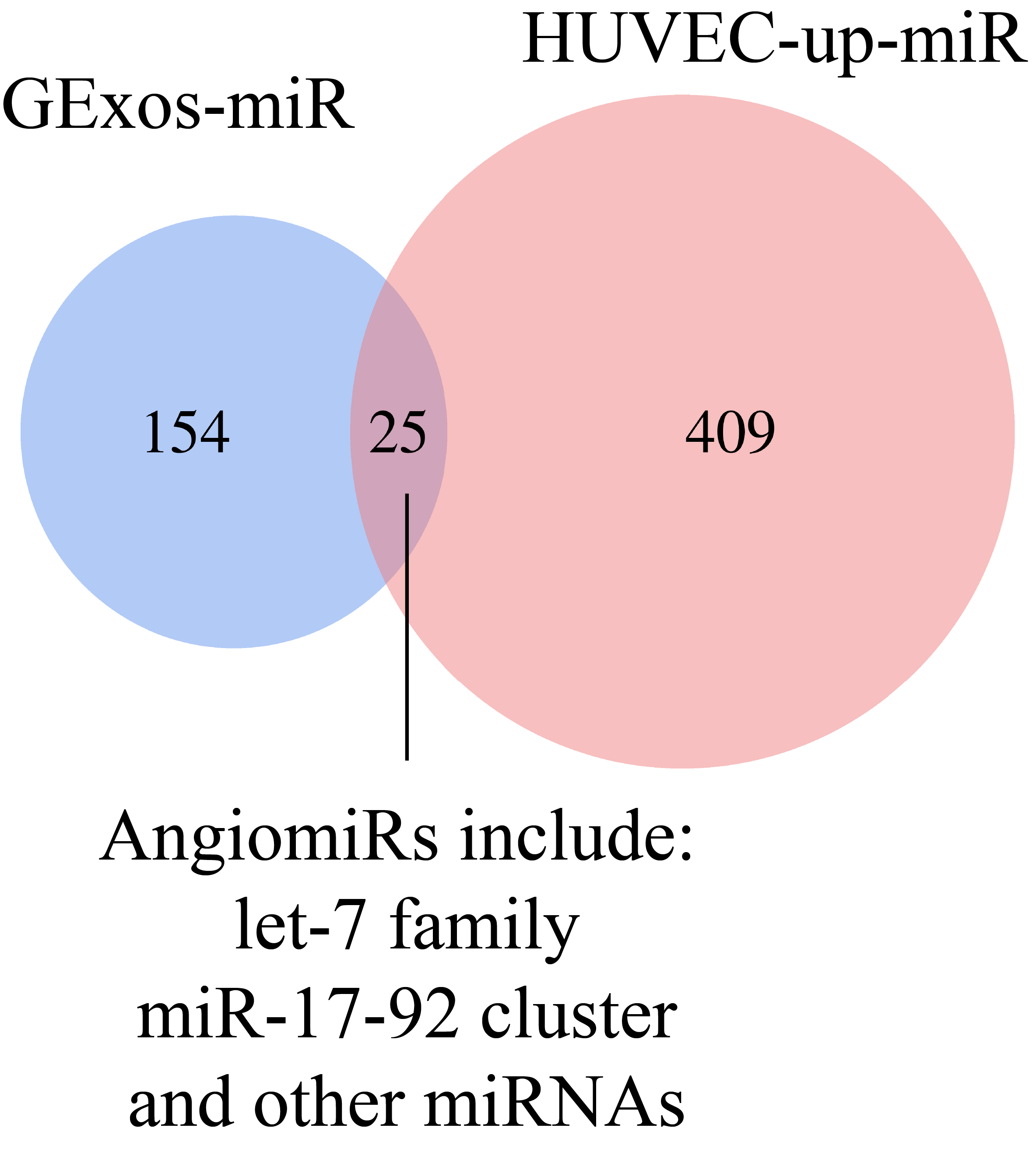


**Figure S8.** Venn diagram of miRNAs delivered by GExos into HUVECs.

**
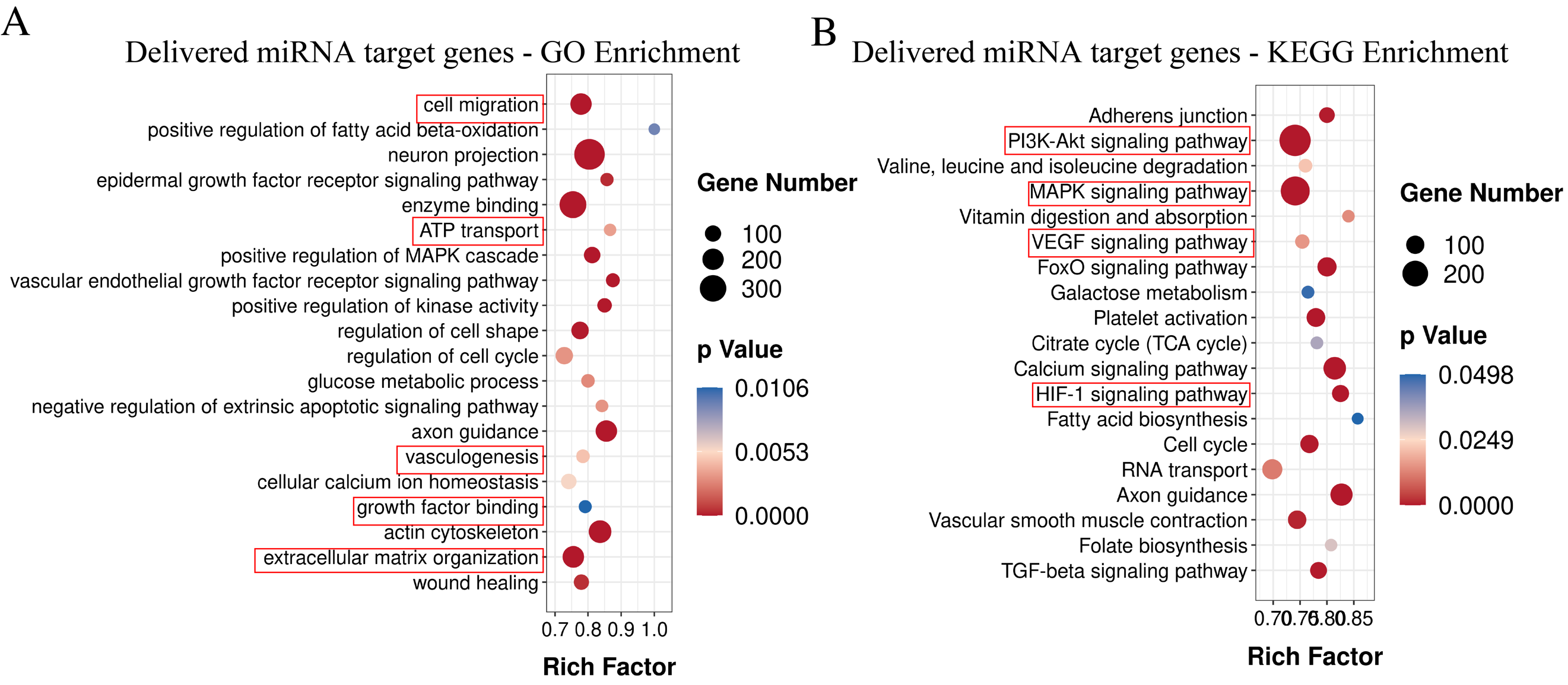
**

**Figure S9.** GO term and KEGG pathway enrichment analysis of the categories enriched in the specific target genes of the GExos-delivered miRNAs.


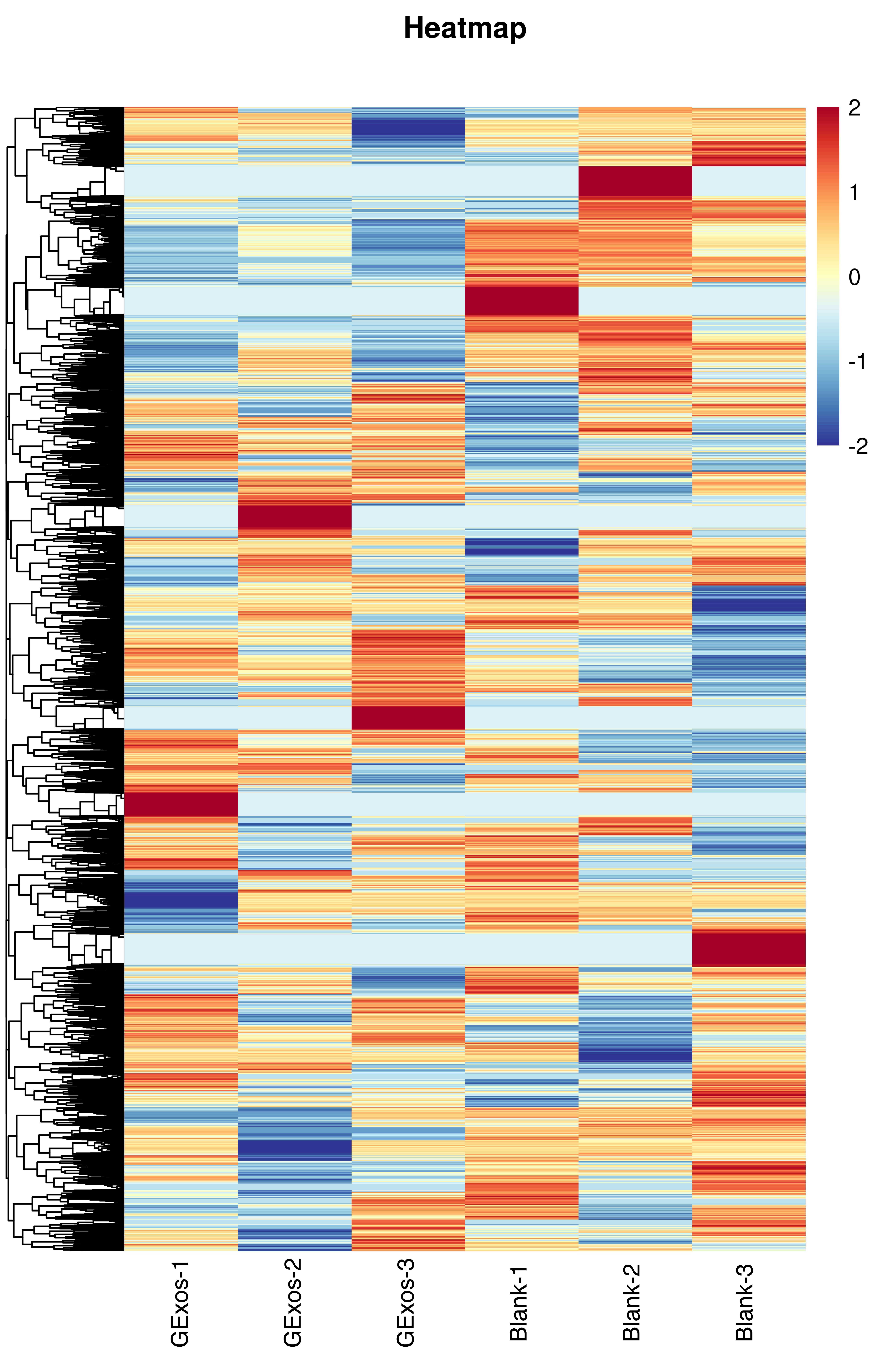


**Figure S10.** Hierarchical clustering of differentially regulated genes identified at q < 0.05 in HUVECs with GExo-treated or not.


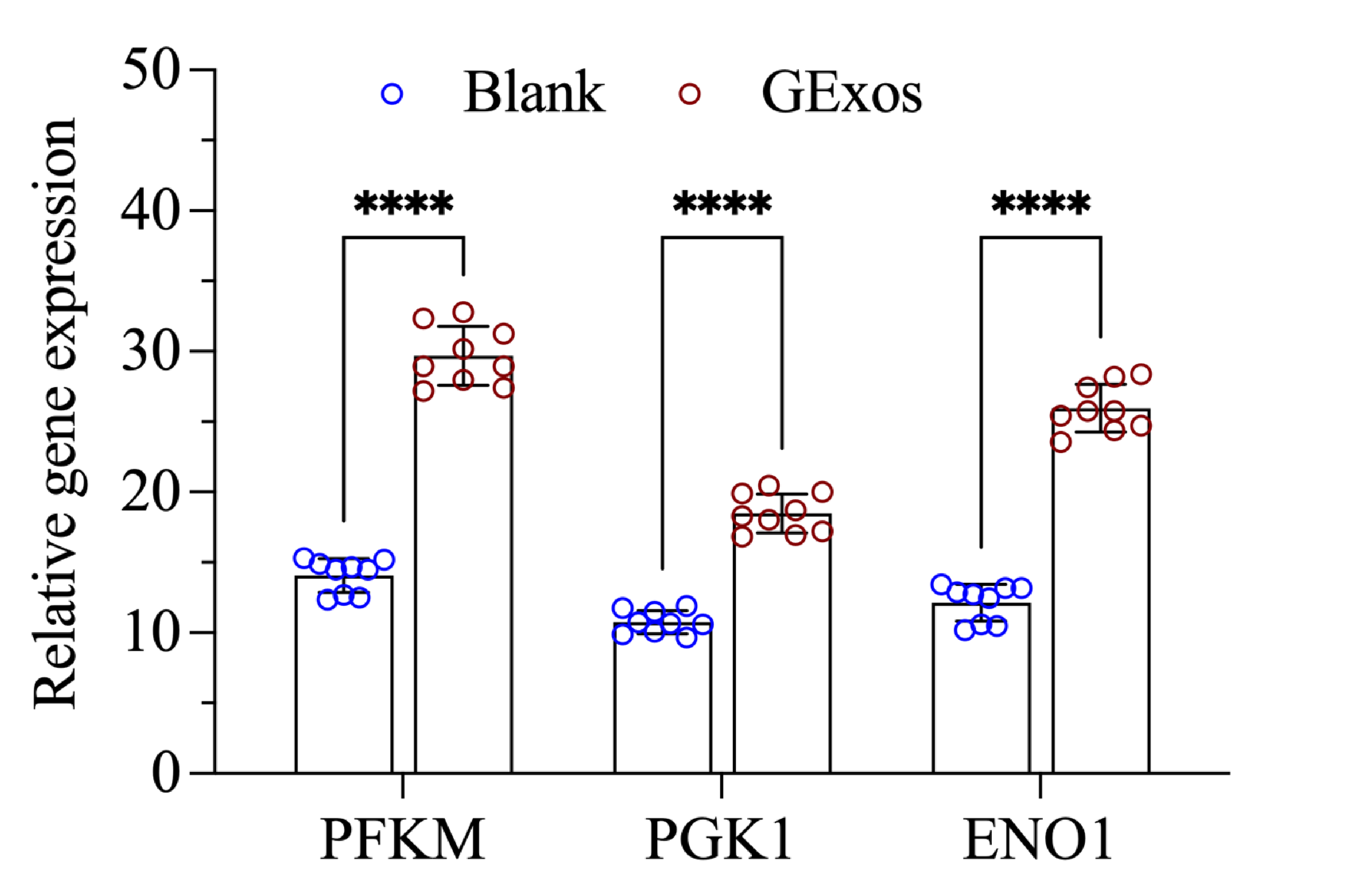


**Figure S11.** Quantification of RT-qPCR validation of PFKM, PGK1, and ENO1.


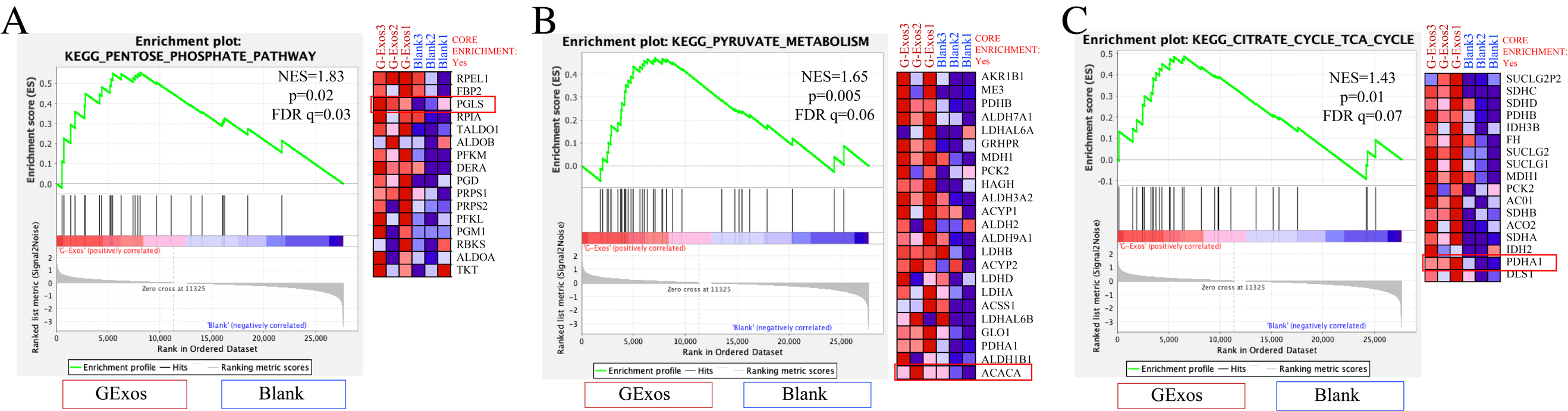


**Figure S12**. Enrichment plots from GSEA analyses of genesets for (A) “Pentose phosphate pathway”, (B) “Pyruvate metabolism” and (C) “Citrate cycle TCA cycle”.


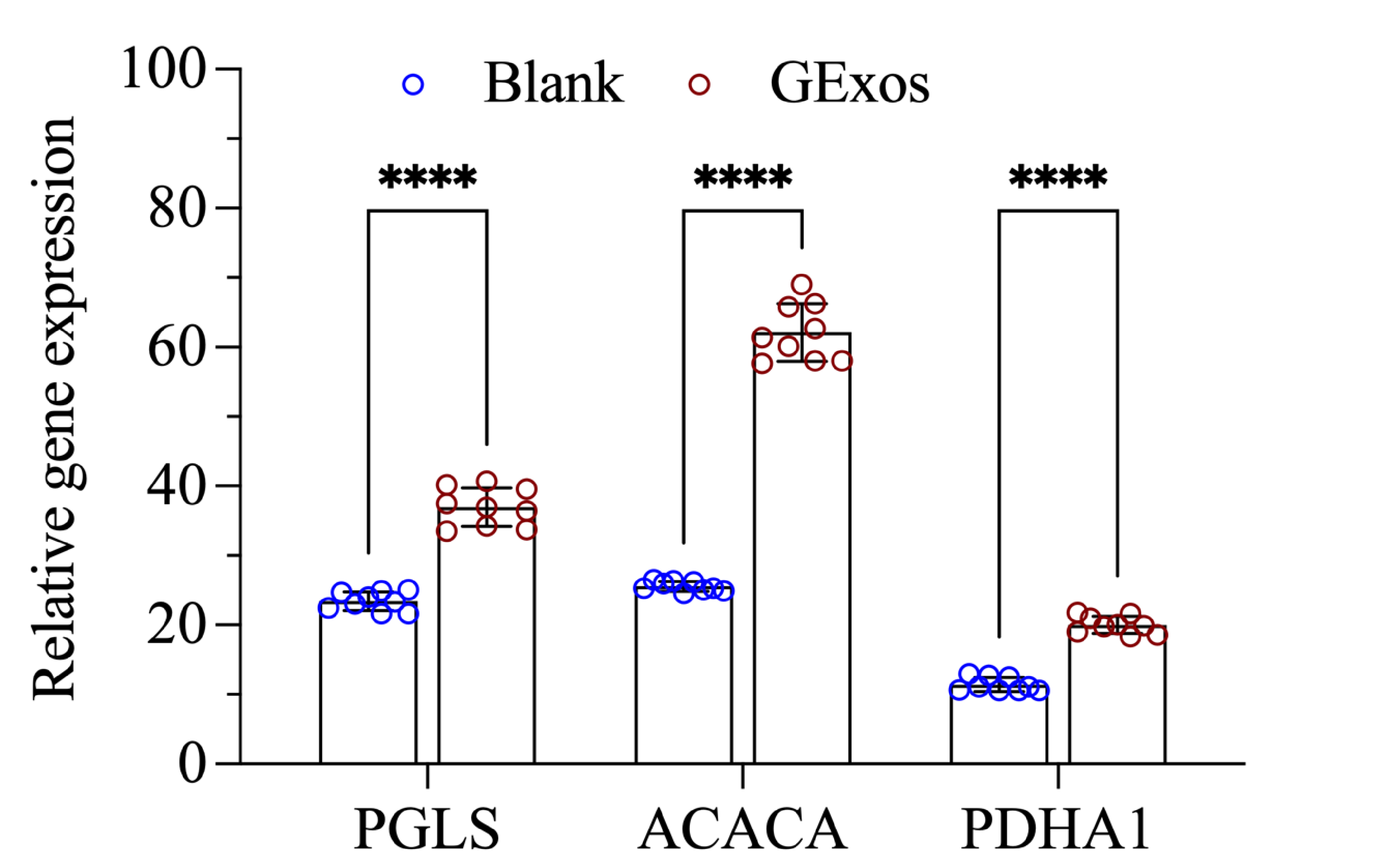


**Figure S13.** Quantification of RT-qPCR validation of PGLS, ACACA, and PDHA1.


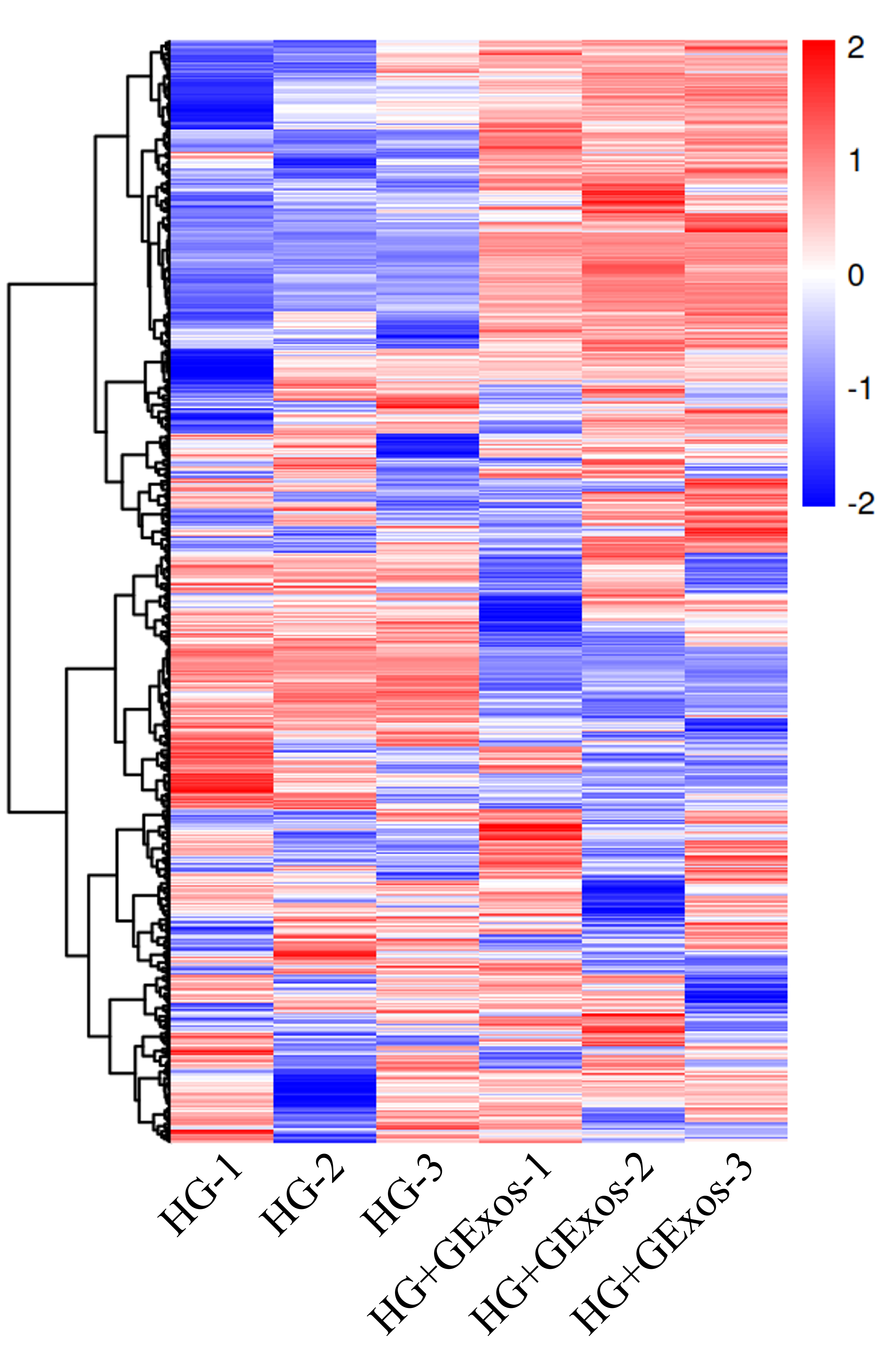


**Figure S14.** Hierarchical clustering of differentially regulated metabolites identified at q < 0.05 in HUVECs with GExo-treated or not.


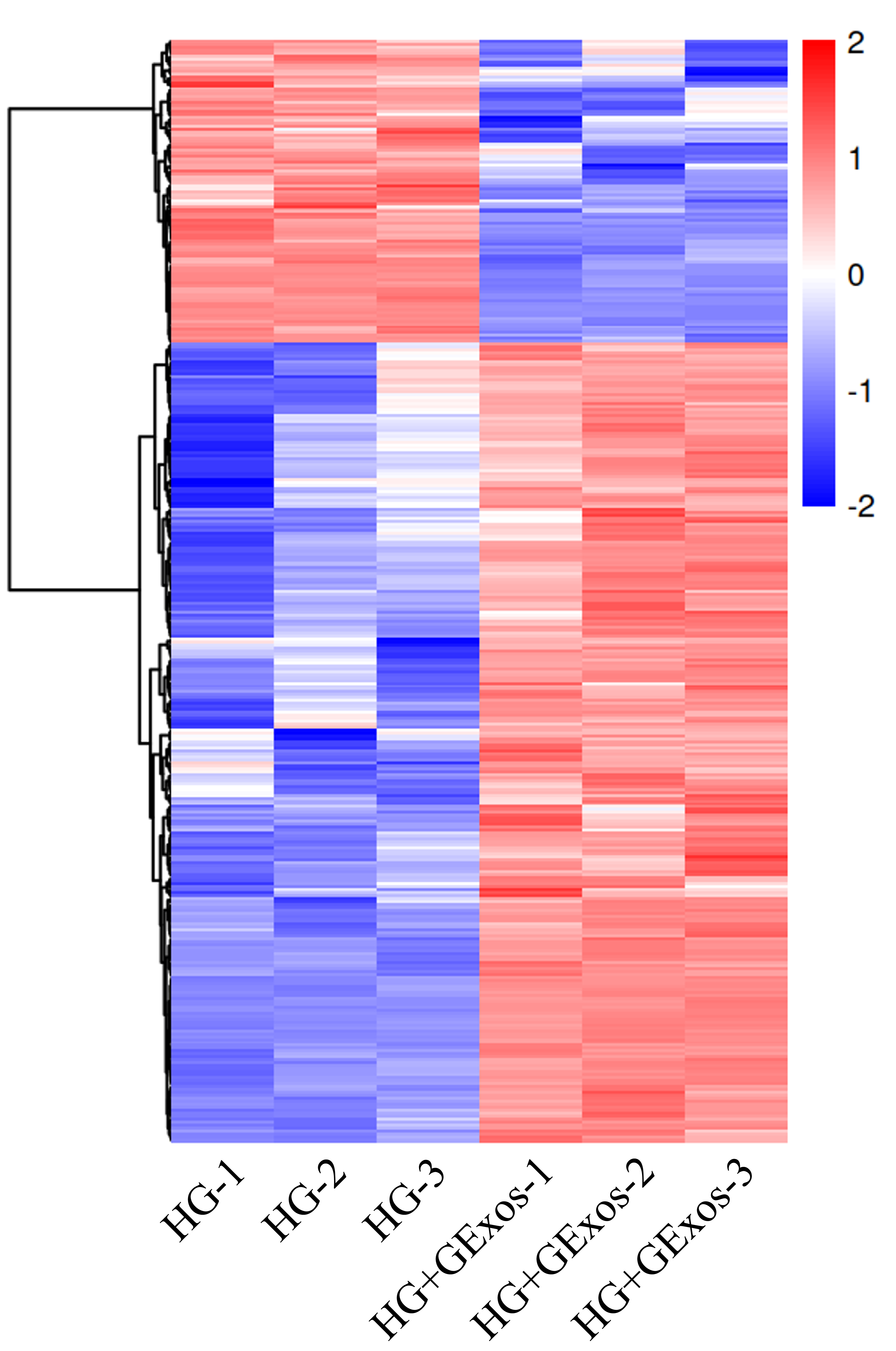


**Figure S15.** Hierarchical clustering of differentially expressed metabolites identified at q < 0.05 in HUVEC cells with HG and HG+GExos cultures.


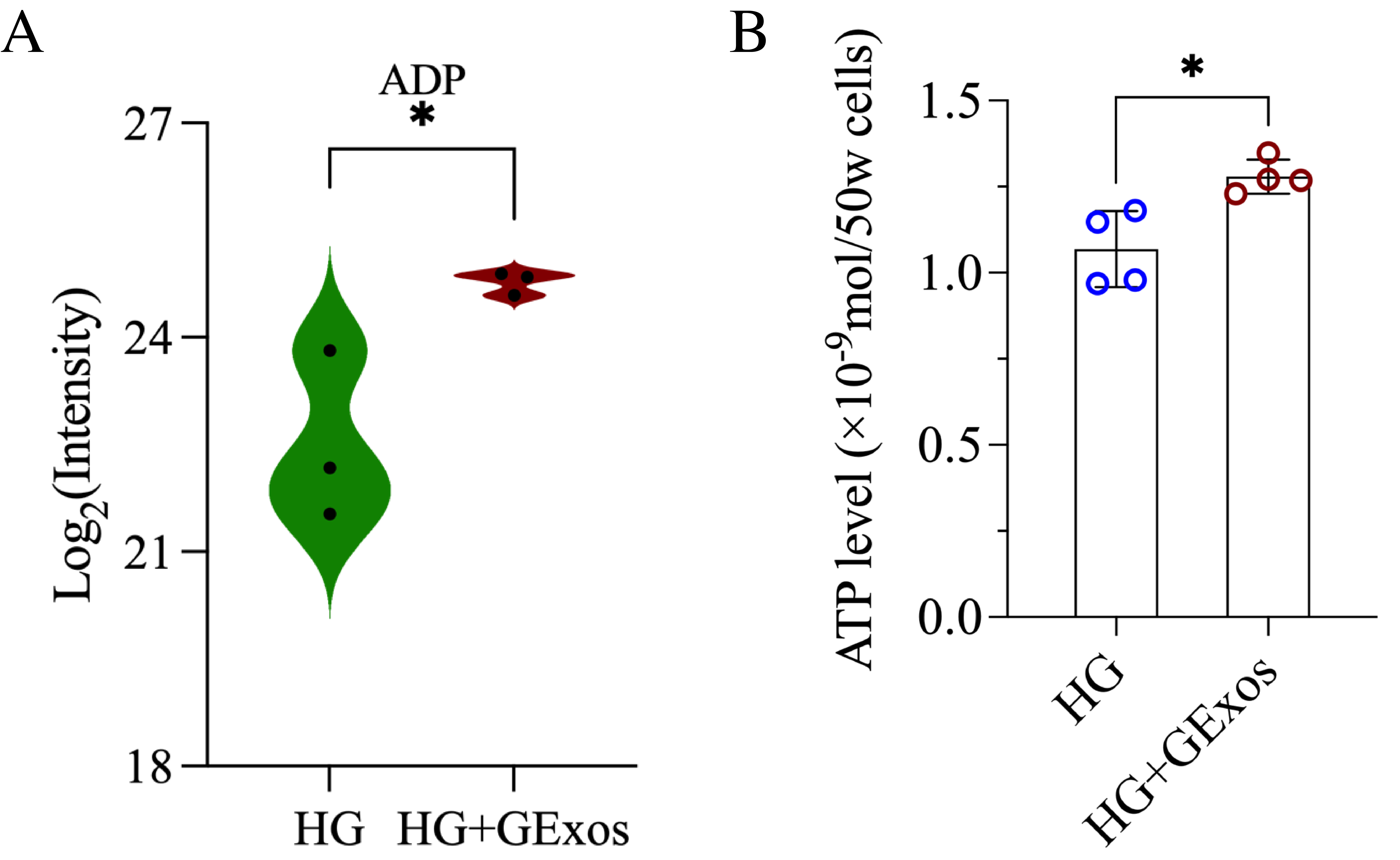


**Figure S16.** Expression levels of ADP (A) and ATP (B) with HG or HG+GExos cultures.


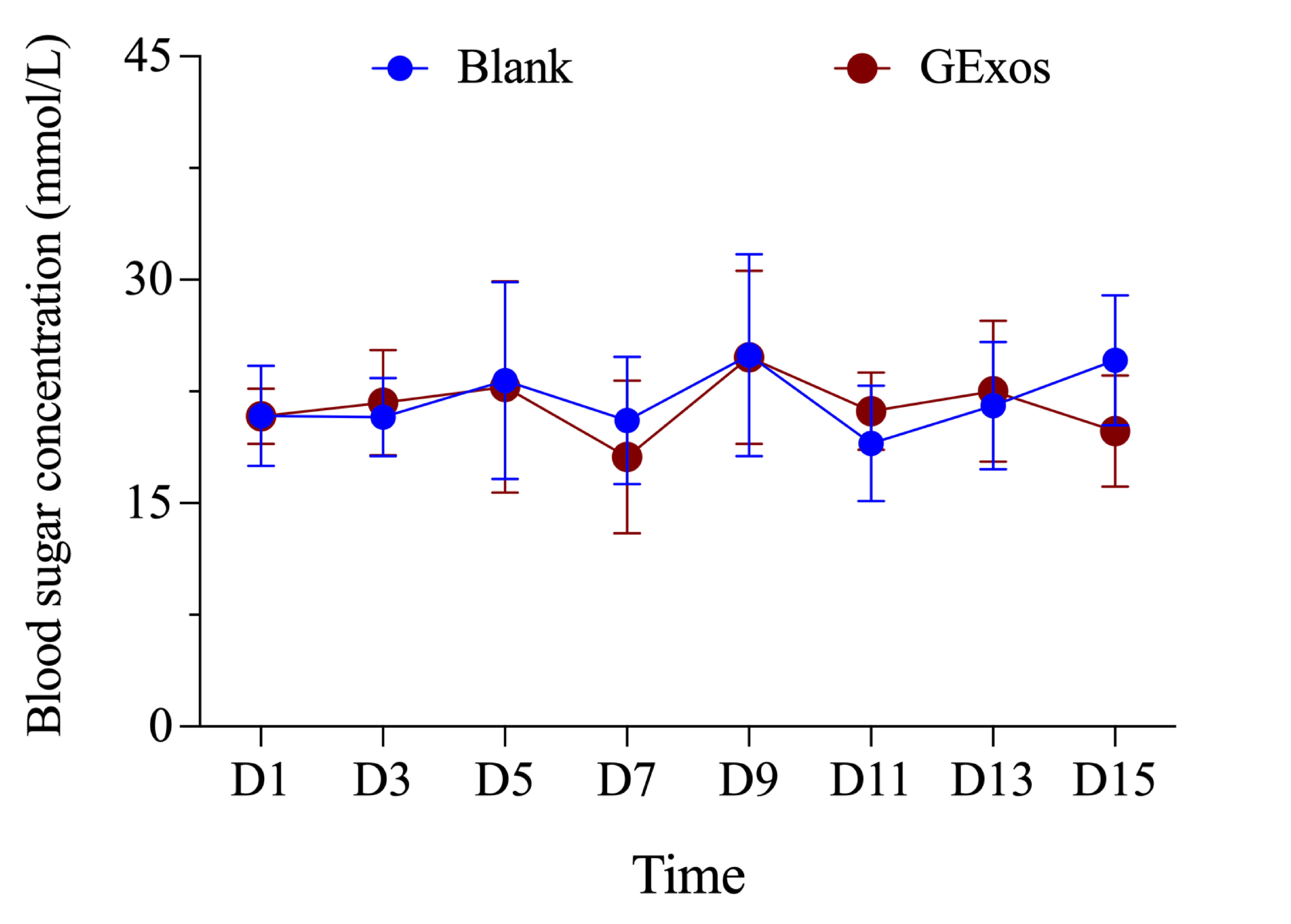


**Figure S17.** Blood sugar concentration (mmol/L) of the *db* mice at different time point in Blank and GExos group.


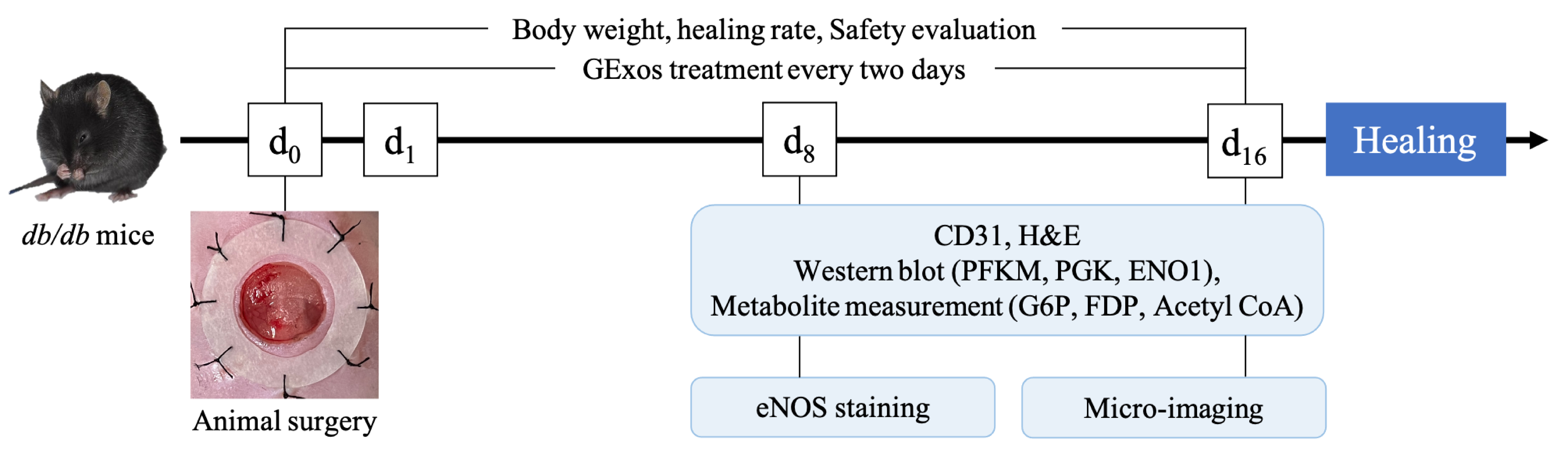


**Figure S18.** Time points scheme of the animal study.


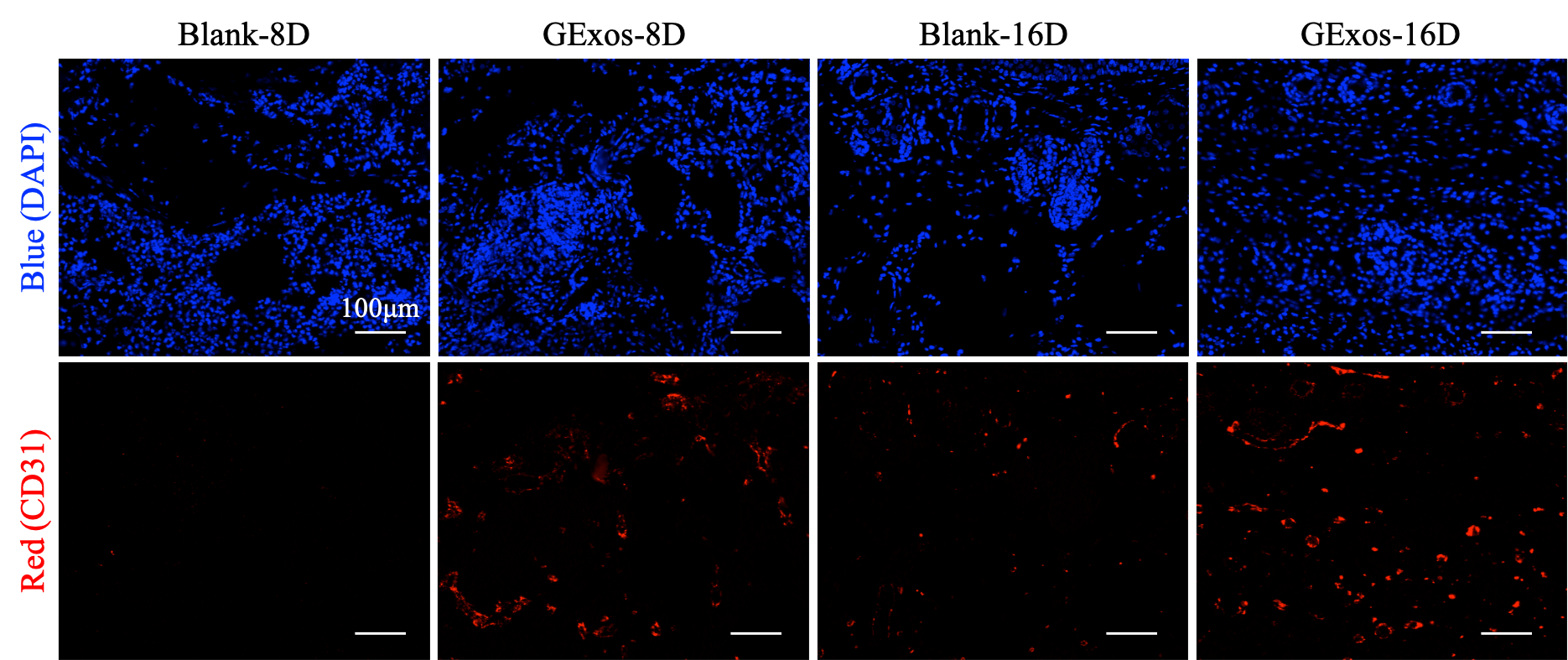


**Figure S19**. The images of CD31 monochromatic fluorescence channels in healed skins on day 8 and 16 post-treatment.


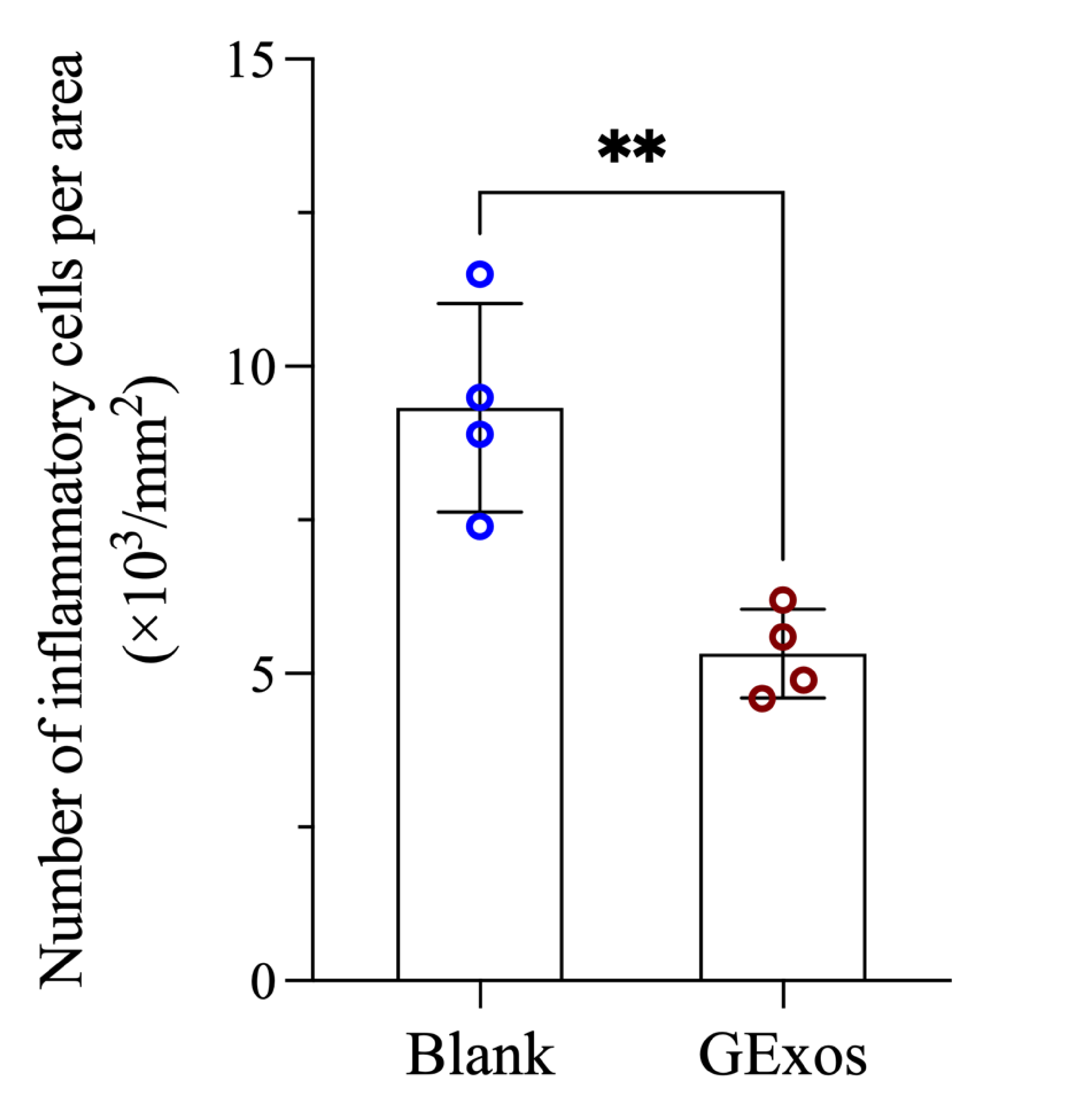


**Figure S20.** Quantitative analysis of inflammatory cell density in Blank and GExos-treated mice groups.


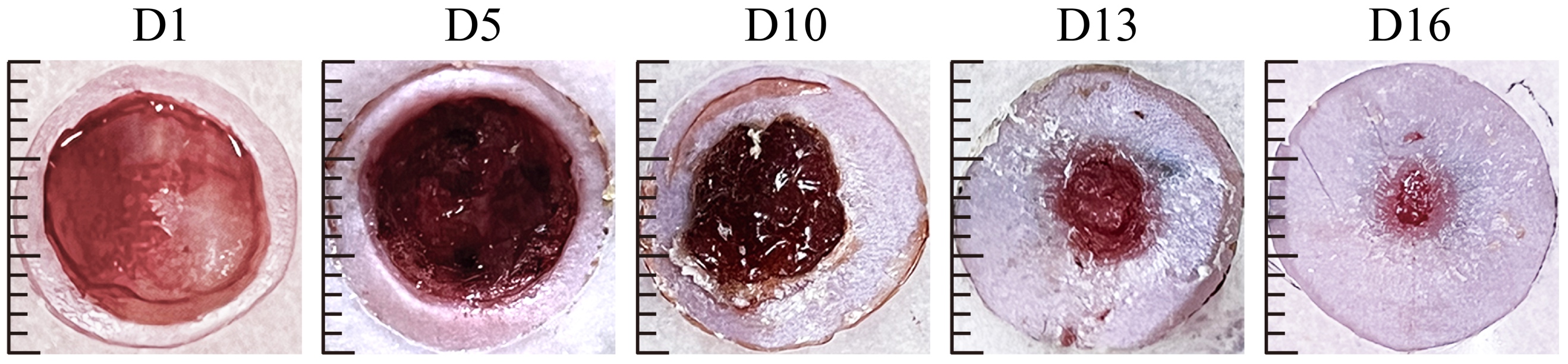


**Figure S21**. Photographs of the wound appearance from day 1 to day 16 post-wounding of the 100 μg·mL^-1^ GExos groups.


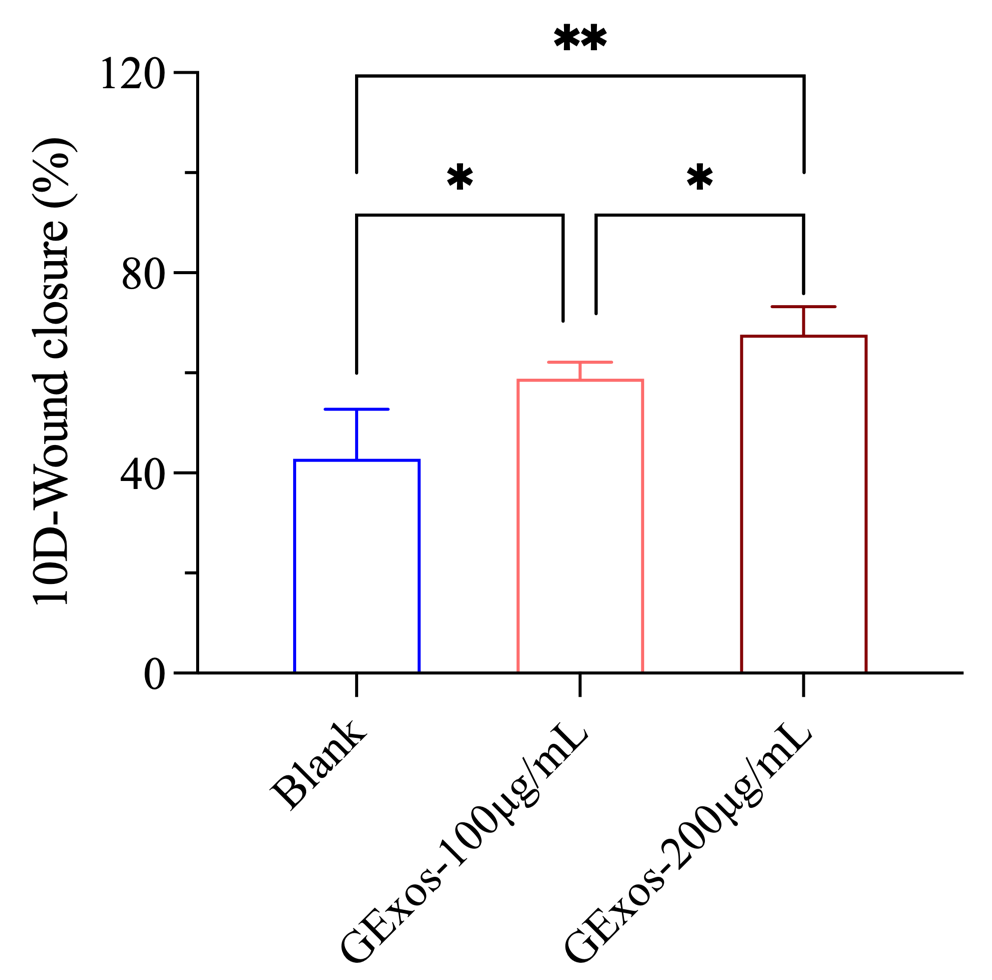


**Figure S22**. Quantitative analysis of wound closure rate of the Blank, 100 μg·mL^-1^ GExos, and 200μg/mL GExos groups on day 10, respectively.


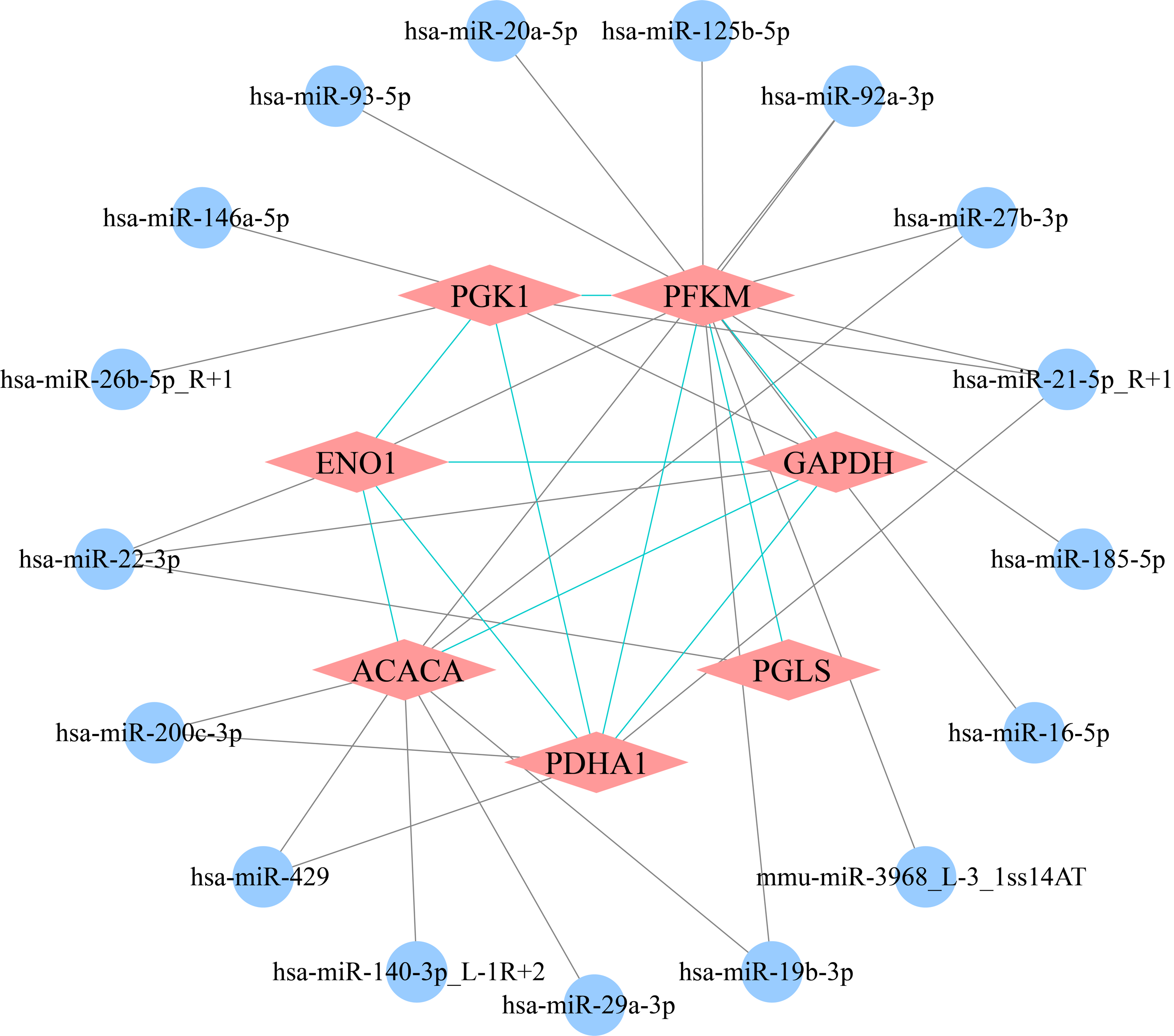


**Figure S23.** The interaction network diagram between the specific mRNAs, and the exogenous miRNAs delivered by GExos into ECs.

**Table S1.** Detection of seven pesticide residues in plant *Ginseng*

| Limit standards | Pesticide test item | Detection value |
| --- | --- | --- |
| ≤0.1ppm | Hexachlorobenzene | 0.005 |
| ≤0.1ppm | Pentachloronitrobenzene (PCNB) | 0.009 |
| ≤0.1ppm | Total benzene hexachloride (BHC) | Not detected |
| ≤0.1ppm | Total dichloro-diphenyl-trichloroethane (DDT) | Not detected |
| ≤0.05ppm | Aldrin metabolized to dieldrin (Aldrin) | Not detected |
| ≤0.05ppm | Heptachlor | Not detected |
| ≤0.1ppm | Chlordane | Not detected |

**Table S2.** Catgories and class of lipid name contained in GExos

| Catgories | classKey | Lipid Name |
| --- | --- | --- |
| Glycerophospholipids  (Phospholipids) | LPC | lysophosphatidylcholine |
|  | PC | phosphatidylcholine |
|  | LPE | lysophosphatidylethanolamine |
|  | PE | phosphatidylethanolamine |
|  | PS | phosphatidylserine |
|  | LPG | lysophosphatidylglycerol |
|  | PG | phosphatidylglycerol |
|  | LPI | lysophosphatidylinositol |
|  | PI | phosphatidylinositol |
|  | PIP | phosphatidylinositol(4)phosphate |
|  | LPA | lysophosphatidic acid |
|  | PA | phosphatidic acid |
|  | CL | Cardiolipin |
| Sphingolipids | SM | sphingomyelin |
|  | LSM | lysosphingomyelin |
|  | phSM | phytosphingosine |
|  | So/SPH | Sphingosine/Sphingosine bases |
|  | CerG1/Hex1Cer | glucocerebroside/Hexosyl ceramide |
|  | CerG2/Hex2Cer | glucocerebroside/Hexosyl ceramide |
|  | CerG3/Hex3Cer | glucocerebroside/Hexosyl ceramide |
|  | CerG2GNAc1 | N-acetylhexosyl ceramide |
|  | ST | Sulfatide (galactosyl ceramide sulfate) |
|  | Cer | Ceramides |
|  | CerP | Ceramides phosphate |
|  | GM3 | Ganglioside, monosialo trihexosyl ceramide |
| Glycerolipids | MG | monoglyceride |
|  | DG | diglyceride |
|  | TG | triglyceride |
| Serol lipids | ChE/CE | Cholesterol Ester |
|  | ZyE | zymosterol |
|  | SiE | Sitosterol ester |
| Prenol lipids | Co | Coenzyme Q |
| Fatty Acyls | OAHFA | (O-acyl)-1-hydroxy fatty acid |
|  | WE | wax exters |
| Saccharolipids | MGMG | Monogalactosylmonoacylglycerol |
|  | MGDG | Monogalactosyldiacylglycerol |
|  | DGMG | Digalactosylmonoacylglycerol |
|  | DGDG | Digalactosyldiacylglycerol |
|  | SQDG | Sulfoquinovosyldiacylglycerol |

**Table S3.** List of miRNAs delivered by GExos into HUVECs.

let-7 family indicate: Number 1-4;

miR-17-92 cluster indicate: Number 6-8, 12, 14;

Other angio-miRs indicate: Number: 9, 10, 15, 24

| Number | Name |
| --- | --- |
| 1 | hsa-let-7f-5p |
| 2 | hsa-let-7e-5p |
| 3 | hsa-let-7i-5p |
| 4 | hsa-let-7b-5p |
| 5 | hsa-miR-16-5p |
| 6 | hsa-miR-17-5p |
| 7 | hsa-miR-19b-3p |
| 8 | hsa-miR-20a-5p |
| 9 | hsa-miR-21-5p |
| 10 | hsa-miR-22-3p |
| 11 | hsa-miR-26b-5p |
| 12 | hsa-miR-27b-3p |
| 13 | hsa-miR-29a-3p |
| 14 | hsa-miR-92a-3p |
| 15 | hsa-miR-93-5p |
| 16 | hsa-miR-125b-5p |
| 17 | hsa-miR-140-3p |
| 18 | hsa-miR-146a-5p |
| 19 | hsa-miR-185-5p |
| 20 | hsa-miR-200c-3p |
| 21 | hsa-miR-222-3p |
| 22 | hsa-miR-429 |
| 23 | hsa-miR-451a |
| 24 | hsa-miR-486-5p |
| 25 | mmu-miR-3968 |
